# Medicinal Plants for Rich People vs. Medicinal Plants for Poor People: A Case Study from the Peruvian Andes

**DOI:** 10.3390/plants10081634

**Published:** 2021-08-09

**Authors:** Fernando Corroto, Jesús Rascón, Elgar Barboza, Manuel J. Macía

**Affiliations:** 1 Instituto de Investigación para el Desarrollo Sustentable de Ceja de Selva, Universidad Nacional Toribio Rodríguez de Mendoza de Amazonas, Calle Universitaria N° 304, Chachapoyas, Amazonas 01001, Peru; fernando.corroto@estudiante.uam.es (F.C.); jesus.rascon@untrm.edu.pe (J.R.); ebarboza@indes-ces.edu.pe (E.B.); 2 Departamento de Biología, Área de Botánica, Universidad Autónoma de Madrid, Calle Darwin 2, ES-28049 Madrid, Spain; 3 Dirección de Desarrollo Tecnológico Agrario, Instituto Nacional de Innovación Agraria, Avenida La Molina N° 1981, Lima 15024, Peru; 4 Centro de Investigación en Biodiversidad y Cambio Global (CIBC-UAM), Universidad Autónoma de Madrid, Calle Darwin 2, ES-28049 Madrid, Spain

**Keywords:** biocultural diversity, ecosystem services, ethnopharmacology, livelihood, medical ethnobotany, medicinal plants market, socio-economic factors, sustainability, urban phytotherapy

## Abstract

Traditional knowledge (TK) of medicinal plants in cities has been poorly studied across different inhabitants’ socioeconomic sectors. We studied the small city of Chachapoyas (~34,000 inhabitants) in the northern Peruvian Andes. We divided the city into three areas according to the socio-economic characteristics of its inhabitants: city center (high), intermediate area (medium), and city periphery (low). We gathered information with 450 participants through semi-structured interviews. Participants of the city periphery showed a higher TK of medicinal plants than participants of the intermediate area, and the latter showed a higher TK than participants of the city center. The acquisition of medicinal plants was mainly through their purchase in markets across the three areas, although it was particularly relevant in the city center (94%). Participants of all socioeconomic levels widely used the same medicinal plants for similar purposes in Chachapoyas, which is likely based on a common Andean culture that unites their TK. However, participants with the lowest socioeconomic level knew and used more plants for different medicinal uses, indicating the necessity of these plants for their livelihoods. City markets with specialized stores that commercialize medicinal plants are key to preserve the good health of poor and rich people living in Andean cities and societies.

## 1. Introduction

Rural migrations usually consist of movements of persons or populations from rural to urban areas [1]. This exodus has existed since cities began to be built thousands of years ago. During the Industrial Revolution period, which occurred at different times worldwide, this process was accelerated by the construction of urban centers surrounded by industrial and productive areas, which resulted in the progressive abandonment of nearby rural areas [2,3].

Today, Latin American populations continue migrating to the cities and rural areas are slowly depopulating [4,5]. Migrants arrive in the cities in a situation of extreme social vulnerability and without economic resources or support networks. Usually, they are installed in peripheral areas that are economically more feasible [6]. In contrast, city centers are occupied by families with greater economic capacities. The price of these downtown properties has increased as business services, financial centers, and official institutions have been set up [7,8]. Thus, the rapid growth of cities in recent decades has changed past peripheral areas to intermediate areas, that have restructured the space progressively with the creation of new peripheric areas [9,10].

Nowadays, Peruvian cities have become areas with very different social strata, related in most cases to the sector of the city where people live, from the city center to the peripheral areas [11]. The different socioeconomic realities that shape the cities show the distinct ways of life of their inhabitants and differences in access to the necessary health services between poor and rich people still persist [12]. In this sense, the economic difficulties faced by the most vulnerable social groups lead them to resort to traditional knowledge (TK) of medicinal plants to protect their health and to fight illnesses [13]. Thus, the use of medicinal plants is essential in cities for economic and social reasons [14,15,16], making migrants and inhabitants from rural origin feel closer to their traditional culture [17,18,19].

Generally, in Peru (and worldwide), people of lower socioeconomic means have higher TK of medicinal plants, because these medicinal resources are crucial for their livelihoods [20,21]. This pattern has been reported widely in the literature [22,23,24]. The socioeconomic level of local people has a direct relationship with the way of gathering these resources from rural areas to cities, where the transformation of the environment and the smaller size of home gardens make access to plants difficult. In this sense, the installation of markets and specialized stores for the sale of medicinal plants is common in Peruvian cities as an indicator of product demand [25,26]).

Some studies in Latin America have reported information on medicinal plant species sold in urban markets [27,28,29,30,31,32]. Other works have focused on medicinal plants used by migrants in different cities worldwide [33,34,35] or compared different population groups based on their socioeconomic characteristics [23,36,37]. But to our knowledge, this is the first study that specifically compares the use of medicinal plants, based on socioeconomic resources of local people, across different areas of the same city.

In this study, we have three objectives. The first objective was to analyze the use of medicinal plants for people with a similar culture and different socioeconomic characteristics, that are living in three areas of the city of Chachapoyas, in the northern Peruvian Andes: (i) city center, (ii) intermediate area, and (iii) city periphery. We hypothesized that people with low economic resources would have greater TK of medicinal plants, which would mainly correspond to the inhabitants of the city periphery [38,39,40]. The second objective was to compare the most important medicinal plant species used and their medical indications across the three areas of the city. We hypothesized that most species and medical indications would be similar across all areas because people come from a common Andean culture [41,42]. The third objective was to compare the mode of acquisition of medicinal plant species across the three areas of the city: (i) collected from the wild, (ii) cultivated in home gardens, family farms, and homes, or (iii) purchased in city markets. We hypothesized that people with higher economic resources would mainly purchase medicinal plants, whereas people with lower economic resources would mainly collect plants from the wild or cultivate them in different ways [43,44,45].

## 2. Results

### 2.1. Distribution of TK of Medicinal Plants across City Areas

The participants of the city of Chachapoyas cited a total of 299 medicinal plant species, belonging to 246 genera and 92 families. They also mentioned 2184 medicinal uses and 5787 use reports. Medicinal plants and uses are shown in Table S1. Specifically, people in the city center cited 175 species, 328 medicinal uses, and 1108 use-reports; in the intermediate area 216 species, 744 medicinal uses, and 1924 use-reports; and in the city periphery 233 species, 1076 medicinal uses, and 2755 use-reports.

**Figure 1:**
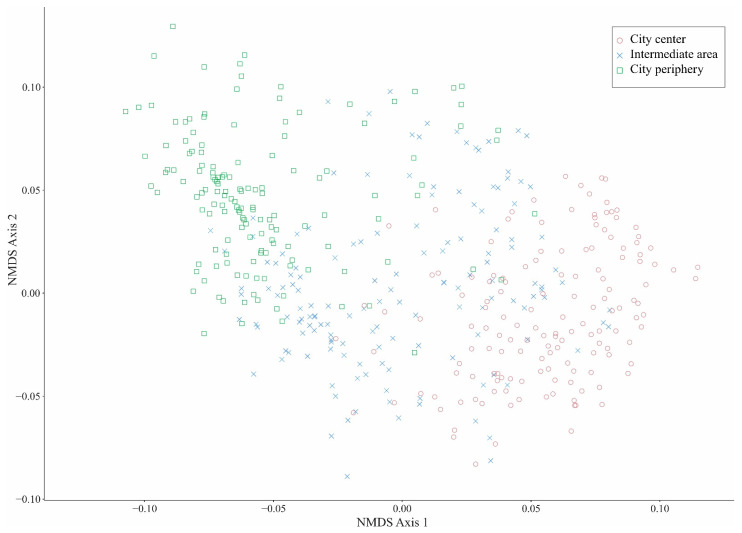
Non-metric multidimensional scaling (NMDS) ordination showing the relative affinity of the 450 participants based on their 21 socioeconomic personal factors across the three areas (city center, intermediate area, and city periphery) in Chachapoyas, Peruvian Andes.

Participants living in the city center and city periphery were separated according to their personal socioeconomic factors, whereas participants living in the intermediate area occupied an intermediate position in a spatial ordination (Figure 1).

**Figure 2:**
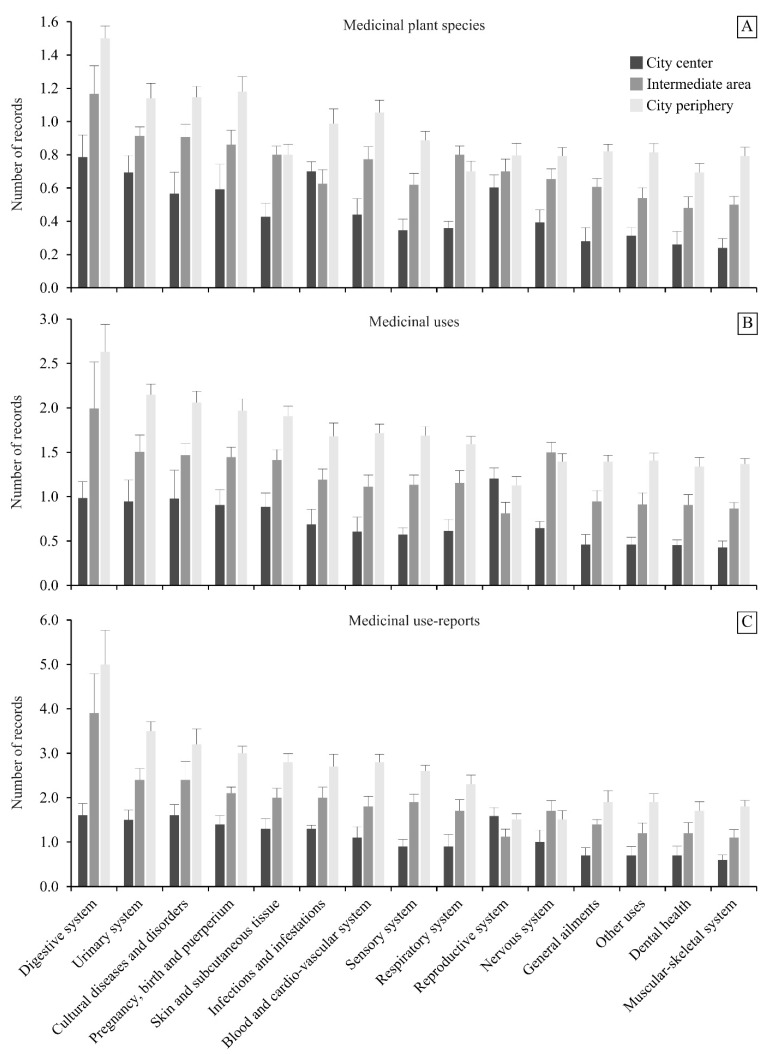
(**A**) Number of useful species (NSP), (**B**) number of medicinal uses (NMU), and (**C**) number of use-reports (NUR) by medicinal categories gathered in 450 interviews with participants from three areas (city center, intermediate area, and city periphery) in the city of Chachapoyas in northern Peruvian Andes.

Overall, participants in the city periphery showed a higher TK of medicinal plants than participants in the two other city areas, based on the three ethnobotanical indicators analyzed for all the medicinal categories (Figure 2). Concerning the ethnobotanical indicator, the number of useful species (NSP), participants in the city periphery cited a higher number of species than participants in the intermediate area, and these participants knew more medicinal species than participants in the city center for all the medicinal categories, with just two exceptions: (i) in the *Infections and infestations category*, participants in the city periphery ranked first, followed by participants in the city center, and finally, participants in the intermediate area; and (ii) in the *Respiratory system category*, participants in the intermediate area ranked first, followed by participants in the city periphery and finally participants in the city center (Figure 2a).

For the two other ethnobotanical indicators, the number of medicinal uses (NMU) and the number of use-reports (NUR), the pattern was the same across the inhabitants of the three city areas: participants in the city periphery clearly knew more medicinal uses and reported more use-reports than participants in the intermediate area, and these participants showed a higher TK of medicinal plants than participants in the city center, with just two exceptions: (i) in the *Reproductive system category*, participants in the city center ranked first, followed by participants in the city periphery, and finally, participants in the intermediate area; and (ii) in the *Nervous system category*, participants in the intermediate area ranked first, followed by participants in the city periphery and finally participants in the city center (Figure 2b,c).

### 2.2. Comparison of the Most Used Medicinal Species and Medical Indications across the City Areas

The 30 most important medicinal plant species used in the three city areas represented 67.4% of the total number of use reports, totaling 42 species (Table 1). 42.8% of these species were reported in all three areas: *Minthostachys mollis* (Benth.) Griseb., *Matricaria recutita* L., *Citrus limon* (L.) Osbeck, *Origanum vulgare* L., *Plantago major* L., *Equisetum bogotense* Kunth, *Malus domestica* Borkh., *Bixa orellana* L., *Ruta chalepensis* L., *Zea mays* L., *Mentha spicata* L., *Aloe vera* (L.) Burm. f., *Chenopodium ambrosioides* L., *Piper acutifolium* Ruiz & Pav., *Erythroxylum coca* Lam., *Solanum lycopersicum* L., *Capsicum pubescens* Ruiz & Pav. and *Tagetes filifolia* Lag. 30.9% of the species were only found in a single area: six species in the city center (*Medicago sativa* L., *Passiflora edulis* Sims., *Phyllanthus niruri* L., *Valeriana adscendens* Turcz., *Verbena litoralis* Kunth and *Croton perspeciosus* Croizat), four species in the intermediate area (*Carica papaya* L., *Musa acuminata* Colla, *Citrus aurantium* L. var. *sinensis* L. and *Stachys arvensis* (L.) L.), and three species in the city periphery (*Spartium junceum* L., *Solanum tuberosum* L. and *Cucurbita maxima* Duchesne). Among the five species with the highest Cultural index in each of the city areas, two of them (*Minthostachys mollis* and *Matricaria recutita*) had the highest Cultural index in all three areas, whereas three species (*Citrus limon*, *Origanum vulgare,* and *Plantago major*) ranked higher in two areas, and four species (*Equisetum bogotense*, *Malus domestica*, *Bixa orellana,* and *Zea mays*) ranked higher in just one city area.

Regarding the status of the most important medicinal plant species, a total of 57.1% were cultivated, whereas 33.3% were native, and 9.5% were naturalized species (Table 1).

The ten most important medical indications in the three city areas represented 58.2% of the total number of use reports, totaling 15 medical indications (Table 2). 46.7% of these medical indications were reported in the three areas, whereas 40.0% were only reported in a single area: three medical indications in the city center (*Prostate disorders*, *Menstruation disorders*, and *Breastfeeding*), two in the intermediate area (*Wounds healing*, and *Fever*), and one in the city periphery (*Burns*). Of the ten most cited medical indications, seven ranked higher in all city areas: *Kidney disorders and diuretic*, *Diarrhea*, *Flu*, *Intestinal parasites*, *Tacsho* (when a person who is going to die manifests itself in another person making him ill), *Insomnia,* and *Birth*.

### 2.3. Acquisition of Medicinal Plant Species across the Three City Areas

**Figure 3:**
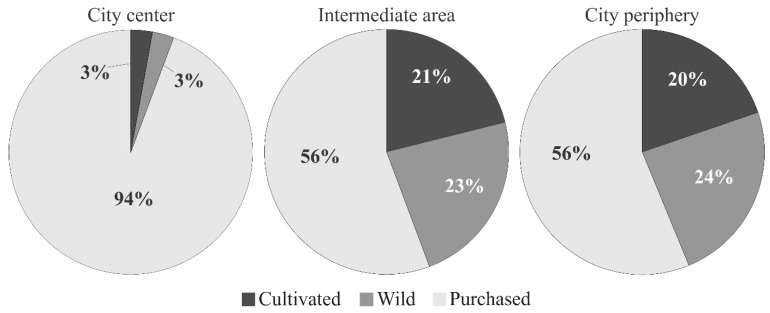
Percentages of medicinal plants acquisition across the three areas of Chachapoyas city from 450 interviews to the local population.

The most common way of acquiring medicinal plants was purchasing them in markets and specialized stores in all three areas of Chachapoyas (Figure 3). This was particularly relevant for the city center where purchases represented 94% of all acquisitions, whereas in both the intermediate area and the city periphery purchases represented 56%. The other ways of acquiring medicinal plants were similar in the intermediate area and the city periphery: 23–24% were harvested from the wild, and 20–21% were cultivated in home gardens, family farms, or homes.

## 3. Discussion

### 3.1. Distribution of the TK of Medicinal Plants in Chachapoyas

Participants of the city periphery clearly showed higher TK of medicinal plants than those of both the intermediate area and the city center, therefore our first hypothesis was accepted. These findings can be explained by economic and cultural factors. First, the high economic cost of health services and medicines limits health access for poor people living mainly in the city periphery [27,46]. Second, residents in the peripheral areas have better access to a natural environment they know and where they can harvest and use medicinal plants [47,48]. The fact that people with lower economic resources use more medicinal plants than people with higher economic resources has been widely reported in the literature [22,23,24], and this study adds to this conclusion.

The most important medical indications for which people use plants in the three city areas are similar to those found in other studies in Latin American cities, with high values in medicinal categories such as *Digestive system* or *Urinary system* [29,49].

The two medicinal categories, *Reproductive system,* and *Nervous system*, for which participants of the intermediate area and the city center had higher TK than participants in the city periphery can be explained from an economic point of view. Some of the most cited medicinal species for the *Reproductive system* (*Jatropha macrantha* Müll. Arg.) and the *Nervous system* (*Valeriana adscendens*) are only sold in specialized stores and city markets at disproportionate prices, and therefore hardly available to the population of the city periphery.

The category *Cultural diseases and disorders* was highly cited in all three city areas, highlighting the great importance that these ailments and diseases have in the culture of these Andean urban societies [27,46]. Cultural diseases and disorders are shown in Table S2. Thus, there is a strong association between certain medicinal species and their medical indications, such as *Minthostachys mollis* to cure *Tacsho*, and *Ruta chalepensis* to alleviate *Malaire*, as reported in past Andean studies [50,51,52].

### 3.2. Cultural Significance of Medicinal Plants in Chachapoyas

Nearly 43% of the species with the highest cultural importance were shared across the three areas of Chachapoyas, and they accounted for more than 67% of the use reports, which also verified our second hypothesis at the species level. A significant part of these species were not native to Peru but are widely used because they are easily cultivated in home gardens or pots at home, and their market price is low [25]. This was the case for *Matricaria recutita*, *Citrus limon*, *Origanum vulgare*, *Malus domestica,* and *Ruta chalepensis*, to name just a few examples. Similarly, many wild species that showed higher cultural importance, such as *Minthostachys mollis*, *Equisetum bogotense*, *Bixa orellana,* and *Piper acutifolium* were easily available. Our study then confirms that many medicinal plants are widely used and have high cultural importance largely due to their availability and accessibility [53,54,55].

Some species were commonly used for different uses, not exclusively medicinal. Many of them are edible species of frequent consumption such as *Zea mays*, *Solanum lycopersicum*, *Capsicum pubescens,* or *Brassica oleracea* var. *acephala,* which increases their cultural importance through the integration of medicinal and nutritional use [56,57,58]. Some other species, such as *Erythroxylum coca* and *Aloe vera*, are among the most important species across the three city areas due to their versatility to treat ailments and disorders of many medicinal categories. These plants are also among the most cited species in many Andean works, highlighting their medicinal versatility, largely because they are used for indications of cultural diseases and ritual and/or magical indications [27,59].

On the other hand, we found 13 medicinal species of great cultural importance that stood out in only one of the three city areas. For example, in the city center, some species (*Phyllanthus niruri*, *Valeriana adscendens,* and *Croton perspeciosus*) are exclusively bought in herbalist stores in forms that resemble conventional medicine. This explains why the highest number of exclusive species was reported in the city center. These types of stores offer (natural) medicinal remedies at higher prices compared to the same species sold in markets [60,61,62]. The opposite also occurs in the city periphery with some species (*Spartium junceum*) that are only collected from the wild and mainly used by participants in the city periphery. Finally, some other species (e.g., *Carica papaya*, *Musa acuminata,* and *Citrus aurantium* var. *sinensis*) cannot be cultivated close to the city of Chachapoyas due to the harsh environmental conditions; they must be imported from other provinces of the Amazonas Department, which increases their cost in markets and makes them less available to participants with fewer economic resources.

Concerning medical indications, we found that participants used medicinal plants for similar purposes regardless of the city area in which they live, so our hypothesis was also accepted at the medical indication level. These results confirm that culture unites the TK of medicinal plants in the city of Chachapoyas. Similar results have been found in other parts of the world, indicating that a small number of medicinal plant species are widely used by the common population [63]. The most frequently cited medical indications in all three city areas include diseases and ailments of the *Digestive system* and the *Urinary system*, which are also widely reported in earlier Andean studies [64,65,66]. Participants in all three areas widely used medicinal plants to deal with *Tacsho*, an idiosyncratic medical indication of Andean cosmology and indicative of a shared cultural past in this society [67,68].

### 3.3. Different Modes of Acquisition of Medicinal Plants in Chachapoyas

Participants of the city center bought 94% of medicinal plants in city markets, but participants in the city periphery and the intermediate area also bought a significant 56% of the plants they used. This indicates that purchase is the first option for all participants and the most advantageous solution to use medicinal plants, as it has also been reported in past studies [25,69]. However, participants in the intermediate area and the city periphery obtained medicinal plants in similar percentages from the wild and/or cultivated, whereas this was not the case of participants in the city center. Therefore, it seems that participants with greater economic resources have less contact with nature, since they hardly harvest wild medicinal plants nor do they cultivate them. In addition, the remoteness of the rural environment from the city center, as well as the ecological characteristics of the environment, prevent city center participants from easily accessing these resources in situ [70].

## 4. Materials and Methods

### 4.1. Study Area and City Areas

**Figure 4:**
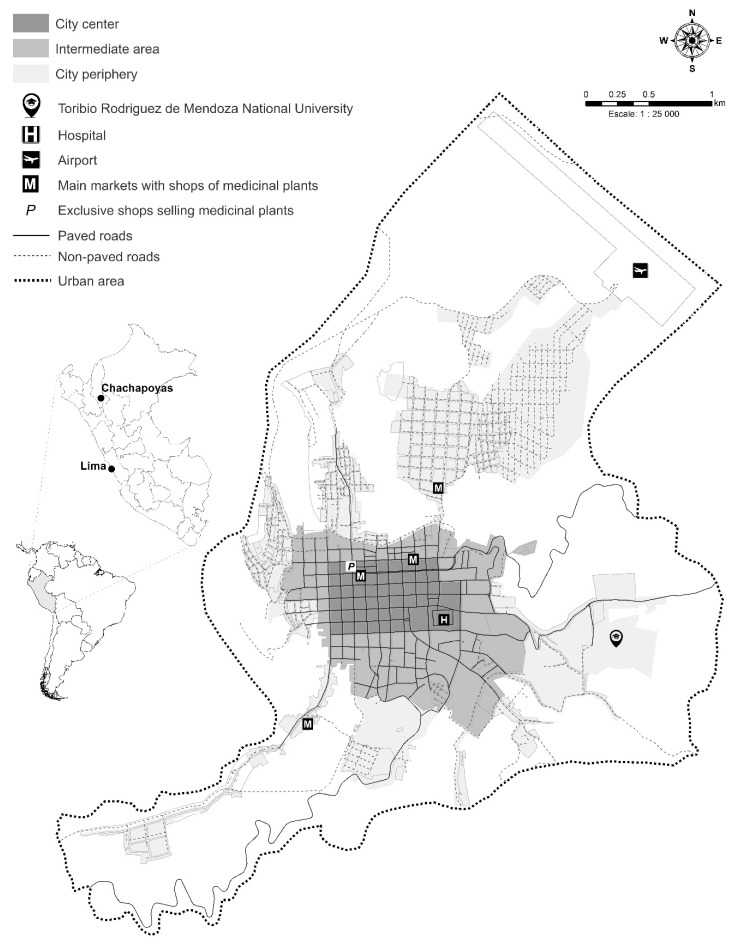
Map of the city of Chachapoyas in the northern Peruvian Andes showing the three areas (city center, intermediate area, and city periphery), where medicinal plants data were gathered from 450 participants.

The city of Chachapoyas (6°13′45.84″ S; 77°52′20.47″ W) is the capital of the Amazonas Department. It is located in the northeastern Peruvian Andes, at 2483 m above sea level, and more than 1200 km apart from Lima (Figure 4). The average annual temperature is 16 °C, the average annual precipitation is around 800 mm, with an average relative humidity of 74%. It has a marked climatic seasonality with the alternance of a rainy season from November to April and a dry season from May to October [71,72].

In the past, it was the most important city of the Chachapoyas human group that was established in the Peruvian Andes between the eighth and fifteenth centuries [73]. Chachapoyas healers were famous in the region because of their use of local plants. Currently, the city of Chachapoyas obtains most of its economic resources from agriculture and livestock in the nearby [74,75].

Chachapoyas city has grown rapidly in the last four decades, increasing from 11,853 inhabitants in the year 1981 to 33,293 inhabitants in 2017 [76]. This growth has occurred through non-uniform population settlements, mainly established in the northern and southeastern parts of the city (Figure 4). The last urban plan of the city classified Chachapoyas in three large areas: city center (~6000 inhabitants), intermediate area (~14,000), and city periphery (~14,000) [77]. In this study, we follow this classification. The most important political and economic official institutions, together with the most significant social and cultural attractions, are established in the city center. The city periphery encompasses all the latest population settlements that started in 2004 during a large migratory period. Finally, the delimitation of the intermediate area, which corresponds to the area between the two other city areas, is based on the decrease in Peru’s internal migration rate in the Amazonas Department between 2002 and 2007 [78].

### 4.2. Data Collection

We identified people’s socioeconomic level based on their place of residence in each city area, respectively: we hypothesized that residents of the city center would have the highest socioeconomic level, residents of the city periphery would have the lowest socioeconomic level, and residents of the intermediate area would have a medium socioeconomic level.

To gather information on the uses of medicinal plants in Chachapoyas city, we conducted 150 semi-structured interviews in each of the three areas, totaling 450 participants. We went through all the streets of each of the city areas looking for participants. The participants were selected according to their place of residence across the three city areas. We interviewed one person per house and family unit agreeing to collaborate. Participants were homogeneously distributed among the streets that make up each area. We sought a balance in terms of gender and age, dividing the interviews equally between men and women, and distributing them into five age groups: 18–30, 31–40, 41–50, 51–60, and over 60 years. The interviews consisted of two parts: (i) a semi-structured interview to gather information of medicinal plants associated with medical indications, and (ii) a structured interview to obtain personal objective information for 21 socioeconomic factors for all participants (Table 3). Interviews were conducted between September 2017 and May 2018 through visits to their homes.

Specimens of the medicinal species were collected in the field, in the home gardens of nine participants, and purchased in the city markets. All vouchers were deposited at the Truxillense Herbarium (HUT) (FC682-FC926) and at the Universidad Nacional Toribio Rodríguez de Mendoza (Peru). The scientific names followed *The Plant List* [79] and the family taxonomic classification followed the *Angiosperm Phylogeny Group* [80].

### 4.3. Data Analysis

The medicinal uses reported during the interviews were classified into 18 categories following international standards [81]. Additionally, we included cultural, ritual, or magical diseases based on Macía et al. [82] and Gruca et al. [83]. Three ethnobotanical indicators were used for each participant: (1) the number of medicinal plant species (NSP) reported; (2) the number of medicinal plants uses (NMU), corresponding to the use of a plant part of a given species that is associated with a medicinal category and a specific medical indication; and (3) the number of medicinal plants use-reports (NUR), corresponding to the sum of all different medicinal uses reported for the total number of known species. To evaluate possible differences between the three areas, we used the 15 medicinal categories with the highest number of use reports (100 or more).

To visualize the relative affinity of the participants to the three city areas, we carried out a non-metric multidimensional scaling analysis (NMDS) with all the participants and including the 21 socioeconomic factors. This analysis was performed in R 3.6.3 [84].

To analyze the significance of the medicinal plant species in each of the three areas of Chachapoyas city, we calculated the Cultural Importance Index (CI), following Tardío and Pardo-de-Santayana [85]. CI results from the sum of the use-reports in every medicinal category (*UR_ui_*) mentioned for a species in an area divided by the number of participants (*N*) in that area, according to the following formula, where *u* is the number of medicinal categories for which a species has been cited, and *i* is the number of participants who have cited it:

(1) CIS=∑u=u1uNC∑i=i1iNURuiN

We obtained a value for each of the species in the whole city, and a value for each of the medicinal species in each of the three city areas.

To compare the most important medicinal uses across the three city areas, we counted all medicinal use-reports per medical indication, respectively. Finally, we compared the mode of acquisition of medicinal plants (cultivated, wild, and purchased) across the three areas based on the number of use reports cited for each case.

### 4.4. Ethics Statement

The study was carried out following the Convention on Biological Diversity ethical rules, considering the Bonn guidelines and the Nagoya Protocol [86,87]. A written permit for the approval of the study was obtained from the local authorities of Chachapoyas, as well as from the Regional Government of the Amazonas Department. We asked each of the participants for free, prior, and informed (verbal) consent, indicating (1) that they could stop the interview at any time, and (2) that the data processing would be anonymous. The ethics committee of the Autonomous University of Madrid approved this study (CEI 73-1327 to M.J. Macía).

## 5. Conclusions

Overall, people from different socioeconomic levels living in Chachapoyas city still trust medicinal plants to alleviate and cure different diseases, ailments and disorders. However, people with the lowest socioeconomic resources living in the city periphery showed higher TK of medicinal plants than people with higher socioeconomic resources living in the city center. Participants shared the use of the most important medicinal plant species and associated medical indications across the three city areas, which is based on the existence of a common Andean culture. Today, all people mostly obtain medicinal plants through purchase in markets and specialized stores, particularly in the city center. However, people living in the city periphery and the intermediate area still harvest plants in the wild and/or cultivate them in different ways; their relationship to nature and traditional culture is surely more vivid.

To our knowledge, this is the first study that compares the use of medicinal plants across different areas of the same city and based on the socioeconomic resources of the population. Therefore, the study could be replicated in other cities in Latin America and other countries worldwide where medicinal plants still play a key role in local culture, in order to confirm/reject our results. Our study could also benefit from comparisons with cities of different population sizes, origins, and cultures (including different religions), and even analyzing the impact of foreign migrations that we did not find in Chachapoyas.

Policymakers should consider the importance of the use of medicinal plants in urban societies and the role of specialized stores to improve livelihoods.

## Figures and Tables

**Table 1:** Comparison of the 30 most important medicinal plant species (with voucher numbers) based on the Cultural Importance Index (in bold; see formula in the Methods section) and broken down across three areas of the city of Chachapoyas (Peruvian Andes).

Species	Family	Status	City Center	Intermediate Area	City Periphery	Whole City
*Minthostachys mollis* (Benth.) Griseb. (FC791)	Lamiaceae	Native	**0.40**	**0.75**	**0.92**	0.69
*Matricaria recutita* L. (FC752)	Compositae	Cultivated	**0.33**	**0.80**	**0.89**	0.67
*Citrus limon* (L.) Osbeck (FC888)	Rutaceae	Cultivated	**0.35**	**0.27**	**0.47**	0.36
*Origanum vulgare* L. (FC790)	Lamiaceae	Cultivated	**0.27**	**0.30**	**0.50**	0.36
*Plantago major* L. (FC861)	Plantaginaceae	Naturalized	**0.19**	**0.39**	**0.46**	0.35
*Equisetum bogotense* Kunth (FC775)	Equisetaceae	Native	**0.10**	**0.32**	**0.46**	0.29
*Malus domestica* Borkh. (FC878)	Rosaceae	Cultivated	**0.11**	**0.44**	**0.27**	0.27
*Bixa orellana* L. (FC717)	Bixaceae	Native	**0.24**	**0.23**	**0.30**	0.26
*Ruta chalepensis* L. (FC892)	Rutaceae	Cultivated	**0.22**	**0.17**	**0.35**	0.25
*Zea mays* L. (FC870)	Poaceae	Cultivated	**0.07**	**0.35**	**0.31**	0.24
*Mentha spicata* L. (FC788)	Lamiaceae	Cultivated	**0.17**	**0.22**	**0.32**	0.24
*Aloe vera* (L.) Burm. f. (FC923)	Xanthorrhoeaceae	Cultivated	**0.14**	**0.14**	**0.39**	0.22
*Chenopodium ambrosioides* L. (FC685)	Amaranthaceae	Naturalized	**0.13**	**0.24**	**0.29**	0.22
*Piper acutifolium* Ruiz & Pav. (FC860)	Piperaceae	Native	**0.09**	**0.24**	**0.33**	0.22
*Erythroxylum coca* Lam. (FC776)	Erythroxylaceae	Cultivated	**0.18**	**0.14**	**0.28**	0.20
*Solanum lycopersicum* L. (FC912)	Solanaceae	Cultivated	**0.11**	**0.14**	**0.27**	0.17
*Capsicum pubescens* Ruiz & Pav. (FC903)	Solanaceae	Cultivated	**0.07**	**0.21**	**0.23**	0.17
*Brassica oleracea* L. var. *acephala* DC. (FC720)	Brassicaceae	Cultivated	**0.15**	**0.18**	0.16	0.16
*Eucalyptus globulus* Labill. (FC842)	Myrtaceae	Cultivated	0.06	**0.16**	**0.27**	0.16
*Tagetes filifolia* Lag. (FC736)	Compositae	Native	**0.13**	**0.16**	**0.20**	0.16
*Petroselinum crispus* (Mill.) Fuss (FC701)	Apiaceae	Cultivated	**0.13**	0.11	**0.24**	0.16
*Desmodium molliculum* (Kunth) DC. (FC815)	Leguminosae	Native	0.03	**0.19**	**0.25**	0.16
*Syzygium aromaticum* (L.) Merr. & L.M. Perry (FC841)	Myrtaceae	Cultivated	0.05	**0.20**	**0.21**	0.15
*Carica papaya* L. (FC729)	Caricaceae	Cultivated	0.06	**0.26**	0.12	0.15
*Musa acuminata* Colla (FC839)	Musaceae	Cultivated	0.04	**0.27**	0.13	0.15
*Cyclanthera pedata* (L.) Schard. (FC768)	Cucurbitaceae	Native	0.01	**0.16**	**0.25**	0.14
*Citrus aurantiifolia* Risso (FC887)	Rutaceae	Cultivated	0.04	**0.14**	**0.22**	0.13
*Apium graveolens* L. (FC698)	Apiaceae	Cultivated	**0.07**	0.12	**0.20**	0.13
*Daucus carota* L. (FC704)	Apiaceae	Cultivated	**0.09**	0.11	**0.19**	0.13
*Ullucus tuberosus* Caldas (FC713)	Basellaceae	Native	**0.08**	**0.15**	0.15	0.13
*Alternanthera mexicana* Moq. (FC684)	Amaranthaceae	Native	**0.11**	**0.13**	0.13	0.12
*Citrus aurantium* L. var. *sinensis* L. (FC890)	Rutaceae	Cultivated	0.06	**0.19**	0.11	0.12
*Spartium junceum* L. (FC816)	Leguminosae	Naturalized	0.03	0.09	**0.23**	0.12
*Medicago sativa* L. (FC802)	Leguminosae	Cultivated	**0.11**	0.06	0.17	0.11
*Passiflora edulis* Sims. (FC853)	Passifloraceae	Cultivated	**0.09**	0.12	0.13	0.11
*Phyllanthus niruri* L. (FC856)	Phyllanthaceae	Native	**0.08**	0.09	0.15	0.11
*Stachys arvensis* (L.) L. (FC796)	Lamiaceae	Native	0.03	**0.13**	0.16	0.11
*Solanum tuberosum* L. (FC910)	Solanaceae	Naturalized	0.05	0.06	**0.20**	0.10
*Valeriana adscendens* Turcz. (FC727)	Caprifoliaceae	Native	**0.07**	0.07	0.16	0.10
*Cucurbita maxima* Duchesne (FC773)	Cucurbitaceae	Cultivated	0.03	0.06	**0.19**	0.09
*Verbena litoralis* Kunth (FC920)	Verbenaceae	Native	**0.07**	0.10	0.11	0.09
*Croton perspeciosus* Croizat (No voucher)	Euphorbiaceae	Native	**0.09**	0.03	0.13	0.08

**Table 2:** Comparison of the 10 most important medicinal uses (in bold) represented by their use-reports (percentages in parentheses) and broken down across the three areas in the city of Chachapoyas (Peruvian Andes).

Medicinal Uses	City Center	Intermediate	City Periphery	Whole City
*Stomach cramps*	31 (2.8)	**161 (8.4)**	**205 (7.4)**	397 (6.9)
*Kidney disorder and diuretic*	**49 (4.4)**	**132 (6.9)**	**211 (7.7)**	392 (6.8)
*Diarrhea*	**46 (4.1)**	**81 (4.2)**	**158 (5.7)**	285 (4.9)
*Flu*	**47 (4.2)**	**95 (4.9)**	**107 (3.9)**	249 (4.3)
*Intestinal parasites*	**55 (5.0)**	**77 (4.0)**	**111 (4.0)**	243 (4.2)
*Tacsho*	**43 (3.9)**	**89 (4.6)**	**106 (3.8)**	238 (4.1)
*Visual disorders*	26 (2.3)	**85 (4.4)**	**121 (4.4)**	232 (4.0)
*Insomnia*	**37 (3.3)**	**77 (4.0)**	**81 (2.9)**	195 (3.4)
*Birth*	**47 (4.2)**	**59 (3.1)**	**84 (3.0)**	190 (3.3)
*Prostate disorders*	**60 (5.4)**	50 (2.6)	73 (2.6)	183 (3.2)
*Menstruation disorders*	**82 (7.4)**	23 (1.2)	56 (2.0)	161 (2.8)
*Wounds healing*	33 (3.0)	**61 (3.2)**	62 (2.2)	156 (2.7)
*Fever*	23 (2.1)	**59 (3.1)**	72 (2.6)	154 (2.7)
*Burns*	27 (2.4)	49 (2.5)	**76 (2.8)**	152 (2.6)
*Breastfeeding*	**37 (3.3)**	48 (2.5)	55 (2.0)	140 (2.4)

**Table 3:** Values assignment for the personal socio-economic factors asked during interviews with 450 participants in the city of Chachapoyas, Peruvian Andes.

Socio-Economic Factors	Variable Classification
Home ownership	(1) Own; (2) Rented
Property quality characteristics	(1) Well maintained; (2) With some defects
Construction materials of the property	(1) Only modern materials; (2) Modern and traditional materials
Water chlorination system	(1) Yes; (2) No
Sewage system	(1) Yes; (2) No
Mobile phone	(1) Yes; (2) No
Radio	(1) Yes; (2) No
Television	(1) Yes; (2) No
Paid TV channels	(1) Yes; (2) No
Internet access	(1) Yes; (2) No
Computer	(1) Yes; (2) No
Printer	(1) Yes; (2) No
Washing machine	(1) Yes; (2) No
Refrigerator	(1) Yes; (2) No
Microwave or oven	(1) Yes; (2) No
Water heater	(1) Yes; (2) No
Off-road vehicle	(1) Yes; (2) No
Conventional car	(1) Yes; (2) No
Motorbike	(1) Yes; (2) No
Bicycle	(1) Yes; (2) No
Cooking fuel	(1) Gas; (2) Wood

## Data Availability

The data presented in this study are available in this article and its Supplementary Material.

## Supplementary Materials

The following are available online at https://www.mdpi.com/article/10.3390/plants10081634/s1, Table S1: Medicinal plants used in the city of Chachapoyas, in the tropical montane forests of northern (Peru). Table S2: Medicinal plants used in the city of Chachapoyas, in the tropical montane forests of northern (Peru).
