# Supplementary material for: Medicinal Plants for Rich People vs. Medicinal Plants for Poor People: A Case Study from the Peruvian Andes
Source: Plants (Basel). 2021 Aug 9;10(8):1634. doi: 10.3390/plants10081634 (PMC8401908; doi:10.3390/plants10081634)
Supplement: Supplementary file 1 [file plants-10-01634-s001.zip › plants-1177136-SI.pdf]

Table S1. Medicinal plants used in the city of Chachapoyas, in the tropical montane forests of northern (Peru).

Scientific Name (voucher)	Vernacular Name	Status	Category	Subcategory	Plant Part	Preparat Ion	Mode of Administration	Informants
**Adoxaceae**								
Sambucus peruviana Kunth (FC682)	Saúco	Cultivated, wild	Cultural diseases and disorders	Susto, espanto	Lf	Dc	Or	1
				Tijte	Bd, Lf	Fs, Ml	Bt, Kn	4
			Digestive system	Diarrhoea	Lf, Sd	If	Or	2
				Laxative	Bd, Lf, St	If	Or	5
			General ailments with unspecific symptoms	Fever	Lf	Fs	Kn	2
				General malaise	Lf	Wm	Pl	1
			Reproductive system and reproductive health	Menopause	Fr	Fs	Or	1
			Skin and subcutaneous tissue	Acne	Lf	Wm	Pl	1
				Itil	Lf	Fs	Kn	1
			Urinary system	Prostate disorders	Lf	If	Or	1
			Metabolic system and nutrition	Weight loss	Lf	If	Or	1
**Amaranthaceae**								
Alternanthera mexicana Moq. (FC684)	Lancetilla de huerta	Cultivated	Blood and cardio-vascular system	Hemorrhoids	Lf	Dc	Bt	1
			Digestive system	Diarrhoea	Lf	Ml	Or	1
			General ailments with unspecific symptoms	Fever	Ep, Lf	If	Bt, Or	13
				Headache	Lf	If	Bt	1
			Infections and infestations	Chickenpox	Ep, Lf	If	Bt, Or	8
				Insect bite	Lf	Dc	Bt	1
			Dental health	Toothache	Lf	Dc	Gg	1
			Sensory system	Visual disorders	Lf	Dc	Bt	2
			Skin and subcutaneous tissue	Wounds, healing	Lf	Dc, Fs, If, Ml	Bt, Kn, Or, Pl	14
				Burns	Lf	Dc	Bt	2
			Urinary system	Kidney disorders, emollient, diuretic	Fr, Lf	Dc, If, Ml	Or, Pl	10
				Prostate disorders	Lf	Dc, If	Or	2
Amaranthus caudatus L.	Kiwicha	Cultivated	Reproductive system and reproductive health	Menopause	Sd	Ml	Or	1
Beta vulgaris L. var. *rapa* Dum. (FC683)	Betarraga	Cultivated	Blood and cardio-vascular system	Anemia	Fr	Dc, Ffs, Ml	Or	37
			Reproductive system and reproductive health	Menopause	Fr	Ml	Or	1
Chenopodium ambrosioides L. (FC685)	Paico	Cultivated, wild	General ailments with unspecific symptoms	Headache	Lf	Dc	Bt	1
			Digestive system	Stomach cramps	Ep, Lf	If	Or	2
				Diarrhoea	Lf	If	Or	1
				Laxative	Lf	If, Ml	Or	3
				Intestinal parasites	Lf	Dc, Fs, If, Ml	Bt, Or	90
			Pregnancy, birth and puerperium	Breastfeeding	Lf	Dc	Or	1
Chenopodium murale L. (FC688)	Shucapaico	Wild	Digestive system	Diarrhoea	Lf	If	Or	1
Chenopodium quinoa Willd. (FC687)	Quinua	Cultivated	Reproductive system and reproductive health	Fertility	Sd	Jc	Or	1
Iresine herbstii Hook (FC686)	Pashquete	Cultivated, wild	Cultural diseases and disorders	Antimonia, gentil, viejo, antiguo	Lf	Fs	Bt	1
				Aire, malaire	Lf	If	Or	1

			General ailments with unspecific symptoms	Fever	Lf	Dc, Fs, If	Bt	4
			Infections and infestations	Chickenpox	Lf	Dc, If	Bt, Or	4
			Pregnancy, birth and puerperium	Postpartum	Lf	Dc, Fs, If	Bt, Or	11
			Skin and subcutaneous tissue	Wounds, healing	Lf	Dc	Bt	1
Spinacia oleracea L. (FC682)	Espinaca	Cultivated	Blood and cardio-vascular system	Anemia	Lf	Dc, Fs, Ml	Or	9
			**Amaryllidaceae**					
Allium cepa L. (FC690)	Cebolla blanca	Cultivated	Blood and cardio-vascular system	Anemia	Ro	Ml	Or	1
			Reproductive system and reproductive health	Aphrodisiac	Ro	Dc	Or	1
			Other uses	Hair loss	Ro	If, Ml	Bt, Pl	2
			Sensory system	Hearing disorders	Ro	Ml	Bt	1
			Skin and subcutaneous tissue	Burns	Ro	Fs, If	Bt, Or, Pl	9
			Respiratory system	Flu	Ro	If	Or	1
Allium fistulosum L. (FC691)	Cebolla china	Cultivated	Infections and infestations	Chickenpest	Ro	Ml	Or	1
				Insect bite	Ro	Ml	Or	1
				Fleas	Ro	Ml, Wm	Kn, Pl	2
			Blood and cardio-vascular system	Anemia	Ro	Fs	Or	1
Allium sativum L. (FC689)	Ajo	Cultivated	Blood and cardio-vascular system	High pressure	Ro	Dc, Fs	Or	2
			Cultural diseases and disorders	Antimonia, gentil, viejo, antiguo	Ro	If	Bt	1
				Tijte	Ro	Ml	Kn	1
				Susto, espanto	Lf	Dc	Bt	1
			Dental health	Toothache	Ro	Dc, Fs, Ml	Bt, Kn, Or	10
			Digestive system	Diarrhoea	Ro	Fs	Or	1
			General ailments with unspecific symptoms	Fever	Ro	Ml	Bt	1
			Infections and infestations	Insect bite	Ro	Ml	Kn	1
				Fleas	Ro	Wm	Kn	1
				UTA, leishmaniasis	Ro	Ml	Kn	1
				Chickenpest	Ro	Ml	Or	1
				Chickenpox	Ro	If	Kn	1
			Skin and subcutaneous tissue	Acne	Ro	Fs, If	Bt, Pl	2
				Wounds, healing	Ro	Ml	Kn	1
				Burns	Ro	Dc	Bt	1
				Feet fungus	Ro	Dc	Bt	1
			**Anacardiaceae**					
Mangifera indica L. (FC694)	Mango	Cultivated	Digestive system	Diarrhoea	Bk, Fr	If	Or	2
				Indigestive	Fr	Fs	Or	1
			Other uses	Cancer	Fr	Fs	Or	1
Mauria heterophylla Kunth	Shimir	Cultivated	Endocrine system	Diabetes	Lf	If	Or	2
			Cultural diseases and disorders	Susto, espanto	Lf	Dc	Or	1
Schinus molle L. (FC695)	Molle	Cultivated, wild	Respiratory system	Flu	Lf	If	Or	1
			Muscular-skeletal system	Rheumatism	Ap, Lf	Fm, Fs, Ml	Bt, Kn, Or, Pl	6
				Joint sprains	Lf	Fm, Wm	Bt, Kn	2
			Infections and infestations	Insect bite	Lf	If	Bt	1
Spondias purpurea L. (FC692)	Ciruela	Cultivated	Digestive system	Laxative	Fr	Fs	Or	2
			Other uses	Cancer	Bd	Dc	Or	1
Toxicodendron striatum (Ruiz & Pav.) Kuntze (FC693)	Itil	Wild	Cultural diseases and disorders	Antimonia, gentil, viejo, antiguo	Lf	If	Bt	1
			Skin and subcutaneous tissue	Itil	Ep, Lf	Fs	Wh	3

**Annonaceae**								
Annona cherimola Mill. (FC696)	Chirimolla	Cultivated	Cultural diseases and disorders	Susto, espanto	Lf	If	Bt	1
			Blood and cardio-vascular system	Anemia	Fr	Fs	Or	2
			Other uses	Cancer	Lf	If	Or	1
				Hair loss	Fr	Fs	Pl	1
			Infections and infestations	Fleas	Sd	Ml	Bt	2
			Muscular-skeletal system	Joint sprains	Lf	If, Wm	Bt, Pl	2
Annona muricata L. (FC697)	Guanábana	Cultivated	Endocrine system	Diabetes	Fr, Lf	Dc, If	Or	2
			Other uses	Cancer	Bd, Bk, Ep, Fr, Lf	Dc, If	Or	21
			Reproductive system and reproductive health	Fertility	Lf	If	Or	1
				Prostate disorders	Bd, Fr, Lf	Dc, Fs, If	Or	11
			Urinary system	Kidney disorders, emollient, diuretic	Lf	If	Or	1
**Apiaceae**								
Apium graveolens L. (FC698)	Apio	Cultivated	Cultural diseases and disorders	Aire, malaire	St	If	Or	1
			Digestive system	Stomach pain	Ep	If	Or	1
				Diarrhoea	Ep. Lf, St	Dc, If, Ml	Or	12
				Stomach infection	Lf	If	Or	1
				Laxative	Lf, St	Dc, If	Or	4
				Stomach cramps	Ep. Lf, St	Dc, If	Or	21
				Intestinal parasites	Lf, St	If	Or	3
			Pregnancy, birth and puerperium	Birth	Ep, Lf, St	Dc, If	Or	4
			Metabolic system and nutrition	Weight loss	Ep. Lf, St	Ddc, If, Ml	Or	8
			Muscular-skeletal system	Joint sprains	Lf	If	Or	1
			Reproductive system and reproductive health	Menstruation disorders	Lf, St	If	Or	3
				Menopause	Lf	Dc	Or	1
			Urinary system	Kidney disorders, emollient, diuretic	Lf, St	Fs, If	Or	2
				Hearing disorders	Ap	Dc	Ew	1
Arracacia peruviana (H. Wolff) Constance (FC705)	Zanahoria del gentil	Wild	Cultural diseases and disorders	Antimonia, gentil, viejo, antiguo	Lf	Dc, Wm	Bt, Kn	2
Arracacia xanthorriza Bancr. (FC702)	Arrachacha blanca	Cultivated	Cultural diseases and disorders	Antimonia, gentil, viejo, antiguo	Ro	If	Bt	1
			Pregnancy, birth and puerperium	Birth	Fr, Lf	Dc, If	Or	2
				Postpartum	Fr	If	Or	1
			Reproductive system and reproductive health	Fertility	Lf	If	Or	1
			Skin and subcutaneous tissue	Itil	Lf	Fs	Bt	1
Coriandrum sativum L. (FC699)	Culantro	Cultivated	Pregnancy, birth and puerperium	Birth	Lf, Sd	If, Ml	Or	2
Daucus carota L. (FC704)	Zanahoria, zanahoria española	Cultivated	Blood and cardio-vascular system	Anemia	Ro	If, Ml	Or	14
			Nervous system and mental health	Insomnia	Ro	Dc	Or	1

			Pregnancy, birth and puerperium	Postpartum	Fr	If	Or	1
				Birth	Fr, Lf	Dc, If	Or	2
			Sensory system	Visual disorders	Lf, Ro	Dc, If, Jc, Ml	Ew	39
			Skin and subcutaneous tissue	Burns	Ro	Ml	Kn	1
Daucus montanus Humb. & Bonpl. ex Schult. (FC703)	Culantrillo, hierba del cuí	Wild	Cultural diseases and disorders	Susto, espanto	Lf	Fs	Bt	1
			Digestive system	Stomach cramps	Lf	If	Or	1
Foeniculum vulgare Mill. (FC700)	Hinojo	Wild	Digestive system	Stomach pain	Lf	If	Or	1
				Stomach cramps	Lf	If	Or	3
				Diarrhoea	Lf	If	Or	2
			Pregnancy, birth and puerperium	Breastfeeding	Lf, Sd	Fs, If	Or	2
			Reproductive system and reproductive health	Menstruation disorders	Ep	Dc	Or	1
			Sensory system	Visual disorders	Lf	Dc	Bt	1
Petroselinum crispus (Mill.) Fuss (FC701)	Perejil	Cultivated	Blood and cardio-vascular system	High pressure	Ep, Lf, Ro	Dc, Fs, If, Jc, Ml	Or	18
				Anemia	Ep, Fr, Lf	Dc, Ml	Or	3
			Digestive system	Stomach cramps	Lf	If	Or	2
				Intestinal parasites	Lf	Dc	Or	2
				Diarrhoea	Lf	Dc	Or	1
			General ailments with unspecific symptoms	Headache	Ro	Ml	Or	1
			Dental health	Toothache	Lf	Fs	Or	1
			Metabolic system and nutrition	Weight loss	Lf	If, Ml	Or	2
			Nervous system and mental health	Insomnia	Ep	If	Or	1
			Other uses	Hair loss	Ro	Ml	Bt	1
			Pregnancy, birth and puerperium	Postpartum	Ep	Ml	Or	1
				Birth	Lf	Dc, If	Or	4
				Abortive	Ro	Dc	Or	2
			Reproductive system and reproductive health	Menstruation disorders	Ep, Lf, Ro	Dc, If, Ml	Or	28
				Menopause	Ep, Lf, Ro	If, Ml	Or	7
			Urinary system	Prostate disorders	Ep, Lf	If	Or	2
			**Apocynaceae**					
Catharanthus roseus (L.) G.Don	Planta chuela	Wild	Cultural diseases and disorders	Antimonia, gentil, viejo, antiguo	Lf	Dc	Bt	1
Metastelma quitense (K. Schum.) Liede (FC706)	Diegolope	Wild	Reproductive system and reproductive health	Menopause	Lf	If	Or	2
			Digestive system	Diarrhoea	Bk	Ml	Or	1
			Sensory system	Hearing disorders	Lf	Dc, Ml, Wm	Bt, Kn	5
			Muscular-skeletal system	Joint sprains	Lf	If	Bt	1
			**Aquifoliaceae**					
Ilex guayusa Loes. (FC707)	Huayusa, guayusa	Cultivated	Metabolic system and nutrition	Weight loss	Lf	Dc	Or	1
			**Arecaceae**					
Cocos nucifera L. (FC709)	Coco	Cultivated	Digestive system	Diarrhoea	Fr	Fs, Ml	Or	2
			Endocrine system	Diabetes	Fr	Fs	Or	1
Mauritia flexuosa L.f. (FC708)	Aguaje	Cultivated	Reproductive system and reproductive health	Menopause	Fr	Jc	Or	2
				Aphrodisiac	Fr	Fs	Or	1
			**Asparagaceae**					
Agave americana L. (FC710)	Penca azul	Wild	Cultural diseases and disorders	Tijte	Lp	Fs, If, Wm	Kn, Or, Pl	6
			Reproductive system and reproductive health	Fertility	Lp	If	Or	1

Furcraea andina Trel. (FC711)	Penca blanca, penca verde de Castilla	Wild	Digestive system	Diarrhoea	Bk	Dc	Or	1
			**Balanophoraceae**					
Corynaea crassa Hook. f. (FC712)	Para para	Wild	Reproductive system and reproductive health	Fertility	Ep	Fm	Or	1
Ullucus tuberosus Caldas (FC713)	Oyuco	Cultivated	Urinary system	Kidney disorders, emollient, diuretic	Ro	If	Or	1
			Pregnancy, birth and puerperium	Birth	Ro	Dc, Fs, If, Jc, Ml	Or	53
				Postpartum	Ro	Dc	Or	1
			Blood and cardio-vascular system	High pressure	Ro	Dc	Or	2
			**Betulaceae**					
Alnus acuminata Kunth (FC714)	Aliso	Wild	Cultural diseases and disorders	Susto, espanto	Lf	Dc	Or	1
				Pulsario	Lf	Fs	Pl	1
				Tacsho	Lf	Fs	Bt	1
			General ailments with unspecific symptoms	General malaise	Lf	Fs, If	Bt, Pl	2
				Fever	Lf	Fs, If	Or, Pl	2
				Joint sprains	Lf	Fs	Pl	2
			Muscular-skeletal system	Rheumatism	Lf	Dc, Ml	Bt, Kn, Or	3
				Hernia	Lf	Ml	Pl	2
			Respiratory system	Flu	Lf	If	Or	1
			**Bignoniaceae**					
Handroanthus serratifolius (Vahl) S.O.Grose	Tahuarí	Wild	Infections and infestations	UTA, leishmaniasis	Ep	Ml	Pl	1
Tecoma stans (L.) Juss. ex Kunth (FC716)	Putquero, pichunche, putquero amarillo	Wild	Cultural diseases and disorders	Pulsario	Lf	Fs	Pl	1
			Pregnancy, birth and puerperium	Birth	Fl, Lf	If	Or	4
Tecoma stans (L.) Juss. ex Kunth var. *sambucifolia* (Kunth) J.R.I.Wood (FC715)	Ciza	Wild	Digestive system	Laxative	St	Ml	Or	1
			**Bixaceae**					
Bixa orellana L. (FC717)	Achiote	Wild	Digestive system	Intestinal parasites	Sd	If	Or	1
				Hepatitis	Sd	Fs	Or	1
			General ailments with unspecific symptoms	Headache	Lf	If	Or	1
			Skin and subcutaneous tissue	Burns	Fr	Ml	Pl	1
			Urinary system	Prostate disorders	Ep, Lf	Dc, If, Ml	Or	108
				Kidney disorders, emollient, diuretic	Lf	If	Or	4
			**Boraginaceae**					
Borago officinalis L. (FC718)	Borraja	Cultivated	Reproductive system and reproductive health	Menopause	Fl	If	Or	1
			Digestive system	Stomach cramps	Lf	If	Or	1
			Respiratory system	Cough	Ep, Fl, Lf	Dc, If	Or	9
				Flu	Lf	Dc, Fs, If	Or	6
Cordia lutea Lam.	Flor de overo	Wild	Digestive system	Hepatitis	Fl	Dc	Or	1
Tiquilia paronychioides (Phil.) A.T. Richardson	Flor de arena	Cultivated	Metabolic system and nutrition	Weight loss	Sd	If	Or	1
			Urinary system	Kidney disorders, emollient, diuretic	Fl	If	Or	1
			**Brassicaceae**					
Brassica oleracea L. var. *acephala* DC. (FC720)	Col, repollo	Cultivated	Blood and cardio-vascular system	Anemia	Lf	Dc	Or	3

			Pregnancy, birth and puerperium	Breastfeeding	Lf	Dc, Fs, If	Or	46
				Birth	Lf	Dc	Or	1
			Sensory system	Visual disorders	Lf	If, Ml	Bt, Ew	3
Brassica oleracea L. var. *italica* Plenck. (FC7190)	Brócoli	Cultivated	Blood and cardio-vascular system	Anemia	Fr	Fs	Or	1
			Other uses	Cancer	Fr	If	Or	1
Erysimum × cheiri (L.) Crantz	Alhelí	Cultivated	Infections and infestations	Chickenpox	Fl	If	Kn	1
Lepidium meyenii Walp. (FC563)	Maca	Cultivated	Blood and cardio-vascular system	Anemia	Fr	Dd, Ml	Or	2
			Pregnancy, birth and puerperium	Birth	Fr	Ml	Or	1
			Reproductive system and reproductive health	Aphrodisiac	Fr, Ro	Dc, If	Or	3
				Menopause	Fr	Dd, Ml	Or	2
Raphanus sativus L. (FC721)	Rabanito	Cultivated	Blood and cardio-vascular system	Anemia	Fr	Fs	Or	1
			Reproductive system and reproductive health	Menstruation disorders	Fr	Ml	Or	1
Rorippa nasturtium-aquaticum (L.) Hayek (FC722)	Berros, verso, versillo	Wild	Blood and cardio-vascular system	Anemia	Lf	Fs	Or	1
			Skin and subcutaneous tissue	Wounds, healing	Lf	Dc	Bt	1
			Urinary system	Kidney disorders, emollient, diuretic	Lf	If	Or	1
				Prostate disorders	Lf	If	Or	1
			**Bromeliaceae**					
Ananas comosus (L.) Merr. (FC723)	Piña	Cultivated	Blood and cardio-vascular system	High pressure	Ec	Fs, Jc, Ml	Or	6
				Hemorrhoids	Ec	Jc	Or	1
			Digestive system	Diarrhoea	Ec	Fs	Or	1
				Stomach pain	Ec	Fs	Or	1
				Indigestive	Ec	Fs	Or	2
			Metabolic system and nutrition	Weight loss	Ec	Fs, Jc, Ml	Or	8
			Urinary system	Kidney disorders, emollient, diuretic	Ec	Dc, Fs, Jc	Or	13
				Prostate disorders	Ec	Dc, Fs, Jc	Or	3
			**Burseraceae**					
Bursera graveolens (Kunth) Triana & Planch. (FC577)	Palo santo	Cultivated	Cultural diseases and disorders	Susto, espanto	Lf	If	Bt	1
			Infections and infestations	Insect bite	Lf	Ml	Pl	1
			**Cactaceae**					
Opuntia ficus-indica (L.) Mill. (FC725)	Tuna	Cultivated	Digestive system	Diarrhoea	Lp	Fs, Ml	Or	7
				Gastric ulcers	Lp	Fs, Ml	Or	2
			Endocrine system	Diabetes	Lp	Ml	Or	4
			Other uses	Cancer	Lp	Fs, Ml	Bt, Or	5
			Reproductive system and reproductive health	Menopause	Lp	Fs, Ml	Bt, Or	2
			Blood and cardio-vascular system	High pressure	Lp	Ml	Or	1
Stenocereus queretaroensis (F.A.C. Weber ex Mathes.) Buxb. (FC725)	Pitajaya	Cultivated	Digestive system	Laxative	Fr	Fs, If, Ml	Or	19
			**Cannaceae**					
Canna indica L. (FC726)	Achira	Cultivated	Digestive system	Laxative	Lf	If	Or	1
			**Capparaceae**					
Capparicordis crotonoides (Kunth) Iltis & Cornejo	Simuro	Wild	General ailments with unspecific symptoms	Headache	Lf	Wm	Pl	1
			**Caprifoliaceae**					
Valeriana adscendens Turcz. (FC727)	Valeriana	Wild	Blood and cardio-vascular system	High pressure	Ro	Dc	Or	1

			Nervous system and mental health	Insomnia	Ep, Lf, Ro	Dc, If	Or	43
			Reproductive system and reproductive health	Menopause	Lf	If	Or	1
**Caricaceae**								
Carica papaya L. (FC729)	Papaya	Cultivated	Blood and cardio-vascular system	Hemorrhoids	Fr	Fs	Or	1
			Cultural diseases and disorders	Tijte	Bk, Lf, Lx, Sd	Fs, Ml, Wm	Bt, Kn, Pl	7
			Digestive system	Intestinal parasites	Sd	Ml	Or	6
				Liver disorders	Fr	Fs	Or	4
				Stomach cramps	Lx	Fs	Or	1
				Laxative	Fr, Sd	Fs, If, Jc, Ml	Or	20
				Diarrhoea	Fr	Fs, Jc	Or	5
				Indigestive	Fr	Fs	Or	18
				Stomach pain	Fr	Fs	Or	4
			Infections and infestations	UTA, leishmaniasis	Lx	Fs	Bt	1
				Fleas	Sd	Ml	Bt	1
			Metabolic system and nutrition	Weight loss	Fr	Jc	Or	1
			Nervous system and mental health	Stress	Fr	Fs	Or	1
				Mental stimulant	Fr	Fs	Or	1
			Sensory system	Visual disorders	Fr	Fs	Or	1
			Urinary system	Kidney disorders, emollient, diuretic	Fr	Fs	Or	1
Vasconcellea microcarpa (Jacq.) A. DC. (FC331)	Maushán	Cultivated	Reproductive system and reproductive health	Vaginal infection	Fr	If	Or	1
**Caryophyllaceae**								
Dianthus caryophyllus L. (FC730)	Clavel	Cultivated	Nervous system and mental health	Sadness	Fl	If	Or	1
**Celastraceae**								
Maytenus macrocarpa (Ruiz & Pav.) Briq. (FC731)	Chuchuasi	Cultivated	Skin and subcutaneous tissue	Acne	Bk	Fm	Or	1
			Digestive system	Stomach cramps	Bk	Fm	Or	1
**Chloranthaceae**								
Hedyosmum scabrum (Ruiz & Pav.) Solms (FC732)	Pirgay, pitillo, huacamuyo	Wild	Pregnancy, birth and puerperium	Breastfeeding	Lf	Dc	Or	1
			Digestive system	Diarrhoea	Lf	Fs	Or	1
**Compositae**								
Acanthoxanthium spinosum (L.) Fourr. (FC748)	Juan Alonso	Wild	Reproductive system and reproductive health	Menstruation disorders	Lf	If	Or	1
Achyrocline alata (Kunth) DC. (FC759)	Huira huira, vira vira, postersatra, fostersacha	Wild	Urinary system	Kidney disorders, emollient, diuretic	Lf	Dc	Or	1
Ageratina exsertovenosa (Klatt) R.M. King & H. Rob. (FC743)	Cruzsacha	Wild	Digestive system	Stomach cramps	Lf	If	Or	1
Ageratina glechonophylla (Less.) R.M. King & H.Rob. (FC746)	Guarme guarme	Wild	Urinary system	Prostate disorders	Lf	If	Or	1
				Kidney disorders, emollient, diuretic	Lf	Dc	Bt, Or	2
Ambrosia peruviana Willd. (FC753)	Marco	Wild	Muscular-skeletal system	Joint sprains	Lf	Wm	Bt	1
			Cultural diseases and disorders	Antimonia, gentil, viejo, antiguo	Lf	If	Pl	1
				Aire, malaire	Lf	If	Bt	1
				Tijte	Lf	If	Or	1
			General ailments with unspecific symptoms	Headache	Lf	If	Bt	1

			Infections and infestations	Lice	Ep	Ml	Kn	1
				Tick bites	Lf	Fs	Pl	1
				Fleas	Lf	Dc, Fs, If, Ml	Bt, Kn	8
			Skin and subcutaneous tissue	Feet fungus	Lf	Dc	Or	1
				Wounds, healing	Lf	Dc	Bt	1
			Pregnancy, birth and puerperium	Abortive	Lf	If	Or	1
			Muscular-skeletal system	Rheumatism	Lf	Dc, If	Bt, Pl	2
Artemisia absinthium L. (FC734)	Ajenjo	Wild	Muscular-skeletal system	Rheumatism	Lf	Fm, If	Bt, Kn	2
				Joint sprains	Lf	Fm	Kn	1
			Cultural diseases and disorders	Tacsho	Lf	Fs	Bt	1
			Skin and subcutaneous tissue	Wounds, healing	Lf	Dc	Bt	1
			Digestive system	Stomach cramps	Lf	If	Or	4
				Diarrhoea	Lf	If	Or	2
				Stomach infection	Lf	If	Or	1
			Infections and infestations	Fleas	Lf	Dc, If	Bt	2
				Chickenpox	Lf	Dc	Bt	1
			Pregnancy, birth and puerperium	Abortive	Lf	If	Or	1
				Postpartum	Ep	Dc	Or	1
			Reproductive system and reproductive health	Menstruation disorders	Ep, Lf	Dc	Or	2
			Urinary system	Kidney disorders, emollient, diuretic	Lf	If	Or	1
Austroeupatorium inulaefolium (Kunth) R.M. King & H. Rob (FC299)	Curmicuna, curomicuna, llashaquirpana	Wild	Cultural diseases and disorders	Susto, espanto	Lf	If	Bt, Or	3
			Skin and subcutaneous tissue	Wounds, healing	Lf	Ml	Bt	1
				Itil	Lf	If	Bt	1
			Digestive system	Stomach cramps	Lf	Dc	Or	1
Baccharis buxifolia (Lam) Pers. (FC761)	Tayanca	Wild	Dental health	Toothache	Lf	Ml	Kn	1
Baccharis genistelloides (Lam.) Pers. (FC762)	Carqueja, tres esquinas	Wild	Cultural diseases and disorders	Susto, espanto	Ep, Lf	Dc, If	Bt, Or	4
			Digestive system	Diarrhoea	Lf	Dc	Or	1
			Reproductive system and reproductive health	Menstruation disorders	Lf	Dc	Or	1
			Urinary system	Kidney disorders, emollient, diuretic	Bd	If	Or	1
Baccharis latifolia (Ruiz & Pav.) Pers. (FC741)	Chillca, camcam	Wild	Cultural diseases and disorders	Susto, espanto	Lf	Ml	Bt	1
				Aire, malaire	Lf	If	Bt	1
				Pulsario	Lf	Fs	Pl	2
				Tacsho	Lf	Fs	Bt	1
			Pregnancy, birth and puerperium	Birth	Lf	Dc, If	Bt, Or	4
			Digestive system	Stomach cramps	Bd, Lf	If	Or	2
				Diarrhoea	Lf	If, Wm	Or, Pl	3
			Sensory system	Hearing disorders	Bd, Lf	Fs, Wm	Bt, Pl	3
			Ritual and magic uses	Curse	Lf	If	Bt	1
			Muscular-skeletal system	Rheumatism	Lf	Fs, Wm	Pl	2
				Joint sprains	Lf	Fs, If, Wm	Bt, Pl	9
			Other uses	Hair loss	Lf	Dc	Bt	1
			Reproductive system and reproductive health	Menstruation disorders	Lf	Wm	Kn	1
			Respiratory system	Cough	Lf	If	Pl	1
				Flu	Lf	If	Or	1
			Dental health	Toothache	Lf	Dc, If	Bt, Gg	2
				Acne	Lf	If	Bt	1

Bidens pilosa L. (FC737)	Cadillo	Wild	Skin and subcutaneous tissue	Burns	Lf	Fs	Pl	1
			Digestive system	Diarrhoea	Ep	If	Bt	1
			Skin and subcutaneous tissue	Wounds, healing	Lf	Dc	Bt	1
			Other uses	Hair loss	Ep, Lf	Dc, Ml	Bt, Pl	2
			Endocrine system	Diabetes	Bk	Dc	Or	1
			Urinary system	Prostate disorders	Bk, Ep, Lf	Dc, If	Or	6
				Kidney disorders, emollient, diuretic	Ep, Lf	Dc, If	Or	5
Calendula officinalis L. (FC300)	Flor del sol	Cultivated	Cultural diseases and disorders	Tijte	Fr	Wm	Pl	1
Cichorium intybus L. (FC733)	Achicoria	Wild	General ailments with unspecific symptoms	Fever	Lf, Ro	Dc, Fs, If, Ml	Bt, Or	5
			Infections and infestations	Chickenpox	Lf	Dc	Bt	1
			Skin and subcutaneous tissue	Wounds, healing	Lf	Dc, If	Bt, Pl	2
			Digestive system	Diarrhoea	Lf	If	Or	2
				Hepatitis	Lf, Ro	If	Or	4
				Liver disorders	Ro	If	Or	1
			Urinary system	Kidney disorders, emollient, diuretic	Lf, Ro	Dc, If	Or	2
Conyza bonariensis (L.) Cronquist (FC751)	Lluilanza	Wild	Cultural diseases and disorders	Susto, espanto	Lf	If	Bt	1
Cynara scolymus L. (FC735)	Alcachofa	Cultivated	Blood and cardio-vascular system	Anemia	Fr	Dc	Or	1
Erysimum × cheiri (L.) Crantz ()	Lalanqui	Wild	Sensory system	Visual disorders	Lf	Dc	Bt	
Gamochaeta americana (Mill.) Wedd. (FC749)	Lechuguilla	Wild	Digestive system	Hepatitis	Lf	Fs	Or	1
			Nervous system and mental health	Insomnia	St	If	Or	1
Lactuca sativa L. (FC324)	Lechuga	Cultivated	Sensory system	Visual disorders	Lf	Fs, If	Pl	2
			Pregnancy, birth and puerperium	Breastfeeding	Lf	Dc, If	Or	2
			Skin and subcutaneous tissue	Burns	Lf	If	Pl	1
			Blood and cardio-vascular system	High pressure	Lf	Fs	Or	2
			Endocrine system	Diabetes	Bd	If	Or	1
			Metabolic system and nutrition	Weight loss	Lf	Fs	Or	1
			Pregnancy, birth and puerperium	Breastfeeding	Ap, Lf	Dc, Jc	Or	3
			Nervous system and mental health	Insomnia	Bd, Lf, St	Dc, If	Or	11
Matricaria recutita L. (FC752)	Manzanilla	Cultivated	Blood and cardio-vascular system	High pressure	Lf	If	Or	2
			Digestive system	Stomach pain	Ep	Dc, If	Or	5
				Stomach cramps	Ep, Lf	Dc, If	Or	79
				Laxative	Lf	If	Or	1
				Diarrhoea	Ep, Lf	Dc, If	Or	7
				Stomach infection	Ep, Lf	Dc, If	Or	5
			General ailments with unspecific symptoms	Fever	Ep, Lf	If	Or	3
			Nervous system and mental health	Insomnia	Ep, Lf	Dc, If	Or	19
				Sadness	Ep	If	Or	1
			Other uses	Cancer	Ep	Dc	Or	1
			Pregnancy, birth and puerperium	Birth	Ep, Lf	If	Or	20
			Reproductive system and reproductive health	Menstruation disorders	Ep, Lf	If	Or	4

				Menopause	Ep, Lf	If	Or	2
			Respiratory system	Flu	Ep, Lf	Dc, If	Or	5
			Sensory system	Visual disorders	Ep, Fl, Lf	Dc, Fs, If	Bt, Ew, Kn	124
				Hearing disorders	Fl	Dc	Bt	1
			Skin and subcutaneous tissue	Acne	Ep, Lf	Dc, Fs, If	Bt, Kn, Or, Vp	28
				Wounds, healing	Lf	Dc	Bt	3
			Urinary system	Prostate disorders	Ep	Dc	Or	1
Monactis jelskii Hieron. (FC755)	Nispasiana	Wild	Cultural diseases and disorders	Susto, espanto	Lf	Fs	Bt	1
Ophryosporus peruvianus R.M. King & H. Rob (FC758)	Rumusauana	Wild	Reproductive system and reproductive health	Menopause	Lf	If	Or	1
			Nervous system and mental health	Sadness	Lf	Ml	Or	1
Pluchea sagittalis Less.	Cuatro esquinas	Cultivated	Skin and subcutaneous tissue	Feet fungus	Lf	Dc	Bt	1
Porophyllum ruderale (Jacq.) Cass. (FC760)	Hierba del gallinazo, shucarruda	Wild	Cultural diseases and disorders	Aire, malaire	Lf	Fs, If	Bt	3
				Susto, espanto	Lf	If	Bt	3
			Nervous system and mental health	Epilepsy	Lf	If	Or	1
Schizotrichia jelskii (Hieron.) "Strother ex Loockerman, B.L.Turner & R.K.Jansen"	Añasquero	Wild	Cultural diseases and disorders	Aire, malaire	Lf	If	Kn	1
Smallanthus glabratus (DC). H. Rob. (FC764)	Yacónsacha	Wild	Cultural diseases and disorders	Pulsario	Lf	If	Or	2
				Susto, espanto	Ep, Lf	If	Or, Pl	2
Smallanthus sonchifolius (Poepp.) H. Rob. (FC763)	Yacón	Cultivated	Blood and cardio-vascular system	High pressure	Ro	If	Or	1
			Reproductive system and reproductive health	Fertility	Ro	If	Or	1
			Endocrine system	Diabetes	Ro	Dc, Fs, If, Jc	Or	20
Sonchus asper (L.) Hill (FC739)	Cashacerraja	Wild	Pregnancy, birth and puerperium	Breastfeeding	Ep	Fs	Wh	1
			Respiratory system	Cough	Lf	If	Or	1
Sonchus oleraceus (L.) L. (FC740)	Cerraja	Wild	Reproductive system and reproductive health	Menstruation disorders	Lf	If, Ml	Or	2
			Pregnancy, birth and puerperium	Postpartum	Lf	If	Bt	1
			General ailments with unspecific symptoms	Fever	Lf	If	Bt, Or	2
Stevia rebaudiana (Bertoni) Bertoni	Estevia	Cultivated	Endocrine system	Diabetes	Lf	Dc, If	Or	16
			Digestive system	Stomach cramps	Lf	If	Or	1
Tagetes elliptica Sm. (FC754)	Marisaccha, maríasacha	Cultivated	Cultural diseases and disorders	Aire, malaire	Lf	If	Bt	2
				Susto, espanto	Lf	If	Bt	1
				Tacsho	Lf	Fs	Bt	1
				Pulsario	Lf	Ml	Pl	1
			General ailments with unspecific symptoms	Fever	Lf	Fm	Kn	1
			Pregnancy, birth and puerperium	Postpartum	Lf	If	Or	1
			Ritual and magic uses	Negative vibes	Lf	If	Bt	1
				Curse	Lf	Ml	Bt	1
				Bring good luck	Lf	Dc	Bt	1
Tagetes erecta L. (FC757)	Ayarrosa, rosa de los muertos	Wild	Sensory system	Visual disorders	Lf	If	Bt	1
			Cultural diseases and disorders	Susto, espanto	Fl, Lf	Dc, Fs, If	Bt, Kn	3
				Tacsho	Ep, Fl, Lf	Fs, If	Bt	4
			Infections and infestations	UTA, leishmaniasis	Lf	Ml	Pl	1
			Digestive system	Hepatitis	Fl	If	Or	1
			Ritual and magic uses	Bring good luck	Fl	Fs	Bt	1

Tagetes filifolia Lag. (FC736)	Anís de sierra	Wild	Pregnancy, birth and puerperium	Birth	Ep, Lf	If	Or	13
			Dental health	Toothache	Lf, Sd	Fs, If	Bt, Or	2
			Blood and cardio-vascular system	High pressure	Ep, Lf	If	Or	2
			Digestive system	Stomach cramps	Ep, Lf	Dc, If	Or	44
				Diarrhoea	Lf	If	Or	4
				Stomach pain	Ep, Lf	If	Or	5
				Hepatitis	Ep	Dc	Or	1
				Stomach infection	Ep, Lf	Dc, If	Or	2
			Reproductive system and reproductive health	Menstruation disorders	Lf	If	Or	1
			Sensory system	Visual disorders	Lf	Dc	Or	1
Tanacetum parthenium (L.) Sch. Bip. (FC738)	Callimanzanilla	Wild	Pregnancy, birth and puerperium	Postpartum	Lf	If	Bt	1
			General ailments with unspecific symptoms	Fever	Lf	Dc, If	Bt	9
				General malaise	Ep	If	Bt	1
			Respiratory system	Flu	Ep, Lf	Dc, Fm, If	Bt, Kn, Or	11
				Cough	Lf	Fm	Or	1
			Digestive system	Hepatitis	Lf	If	Or	1
Taraxacum officinale (L.) Weber ex F.H. Wigg (FC745)	Diente de león, amargón	Wild	Pregnancy, birth and puerperium	Abortive	Lf	Dc	Or	1
			Other uses	Hair loss	Lf	If	Bt	1
			General ailments with unspecific symptoms	Fever	Ep	Dc	Or	2
			Digestive system	Gastric ulcers	Lf	If	Or	2
Tessaria integrifolia Ruiz & Pav. (FC756)	Pájaro bobo	Wild	General ailments with unspecific symptoms	General malaise	Lf	Fs	Bt	1
			Urinary system	Kidney disorders, emollient, diuretic	Lf	Dc	Or	1
Vernonanthura patens (Kunth) H. Rob. (FC742)	Cosomo, cosmo	Wild	Digestive system	Diarrhoea	Lf	If	Or	1
Vernonia scorpioides (Lam.) Pers. (FC747)	Gulgul	Wild	Muscular-skeletal system	Joint sprains	Bk	Fs	Kn	1
			Skin and subcutaneous tissue	UTA, leishmaniasis	Lf	Fs	Pl	1
Zinnia peruviana (L.) L (no voucher specimen)	Chinita y cholito	Cultivated	Reproductive system and reproductive health	Fertility	Fr, Lf	If	Or	4
**Convolvulaceae**								
Ipomoea alba L. (FC765)	Acniuca blanca	Wild	Sensory system	Visual disorders	Ap	Dc	Bt	1
Ipomoea batatas (L.) Lam. (FC766)	Camote	Cultivated	Pregnancy, birth and puerperium	Breastfeeding	Bd, Lf, Lx. Ro	Dc, Fs, If	Or, Pl	9
			Skin and subcutaneous tissue	Feet fungus	Ro	Dc	Bt	1
**Crassulaceae**								
Kalanchoe pinnata (Lam.) Pers. (FC767)	Hoja del aire, pimpinela	Wild	Infections and infestations	Malaria	Lf	If	Or	1
**Cucurbitaceae**								
Citrullus lanatus (Thunb.) Matsum. & Nakai (FC772)	Sandía	Cultivated	Urinary system	Kidney disorders, emollient, diuretic	Fr	Fs	Or	2
			General ailments with unspecific symptoms	Headache	Fr	Fs	Or	1
Cucumis melo L. (FC770)	Melón	Cultivated	Urinary system	Kidney disorders, emollient, diuretic	Fr	Fs	Or	1
Cucumis sativus L. (FC771)	Pepinillo	Cultivated	Blood and cardio-vascular system	High pressure	Fr	Fs, Ml	Or	3
				Hemorrhoids	Fr	Fs, Ml	Kn, Or	2
			Skin and subcutaneous tissue	Acne	Fr	Fs	Bt, Kn, Or, Pl	5
				Burns	Fr	Fs	Bt, Pl	3

			Digestive system	Diarrhoea	Fr	Fs, If	Or	3
			Pregnancy, birth and puerperium	Birth	Fr	Fs	Or	1
			Endocrine system	Diabetes	Fr	Fs	Or	1
			Metabolic system and nutrition	Weight loss	Fr	Fs, If, Ml	Or	6
			Sensory system	Visual disorders	Fr	Fs	Ew	1
Cucurbita ficifolia Bouché (FC769)	Chiclayo, calabaza	Cultivated	Cultural diseases and disorders	Tijte	Lf	Wm	Bt	1
				Antimonia, gentil, viejo, antiguo	Sd	Fs	Bt	1
			Digestive system	Intestinal parasites	Sd	Dc, Ml	Or	2
				Laxative	Sd	Wm	Or	1
			General ailments with unspecific symptoms	Fever	Lf	Dc, Fs, If	Bt	4
			Reproductive system and reproductive health	Fertility	Lf	If	Or	1
			Urinary system	Prostate disorders	Bd	If	Or	1
			Pregnancy, birth and puerperium	Postpartum	Lf	If	Bt	1
				Breastfeeding	Sd	Wm	Or	1
Cucurbita maxima Duchesne (FC773)	Zapallo, zapayo	Cultivated	Cultural diseases and disorders	Antimonia, gentil, viejo, antiguo	Sd	Fs	Bt	1
			Reproductive system and reproductive health	Fertility	Sd	If	Or	1
				Menstruation disorders	Lf	If	Or	1
			Digestive system	Intestinal parasites	Sd, Lf	Dc, Fs, If, Ml, Wm	Or	32
				Laxative	Sd	If, Ml	Or, Pl	5
			Nervous system and mental health	Insomnia	Fr	Fs	Or	1
Cyclanthera pedata (L.) Schard. (FC768)	Caigua, cayua	Cultivated	Endocrine system	Diabetes	Lf	Dc	Or	1
			Pregnancy, birth and puerperium	Abortive	Fr	Dc	Or	1
			Blood and cardio-vascular system	High pressure	Fr	Jc	Or	1
			Cultural diseases and disorders	Antimonia, gentil, viejo, antiguo	Sd	Fs	Bt	1
			Endocrine system	Diabetes	Fr	Ml	Bt, Or, Pl	1
			Sensory system	Hearing disorders	Lf	Jc, Ml, Wm	Or	57
				Visual disorders	Fr, Lf	Fs, Ml	Bt, Ew, Or	5
Sicana odorifera (Vell.) Naudin	Secana	Cultivated, wild	Reproductive system and reproductive health	Menstruation disorders	Fr	Dc	Or	1
				Menopause	Fr, Lf	If	Or	2
			**Cupressaceae**					
Cupressus sempervirens L. (FC774)	Ciprés	Cultivated	Respiratory system	Flu	Lf	If	Or	1
			**Equisetaceae**					
Equisetum bogotense Kunth (FC775)	Cola de caballo	Wild	Digestive system	Stomach cramps	Lf, Ro	Dc	Or	2
				Diarrhoea	Lf	Dc	Or	1
			Dental health	Toothache	Ep	Dc	Gg	1
			Other uses	Cancer	Ep	Dc	Or	1
			Reproductive system and reproductive health	Menstruation disorders	Lf	If	Or	1
				Vaginal infection	Ep	If	Or	1
			Skin and subcutaneous tissue	Wounds, healing	Ep	Dc, If	Bt	3
			Urinary system	Kidney disorders,	Ep, Lf	Dc, If	Or	117

				emollient, diuretic				
				Prostate disorders	Ep, Lf	Dc, If	Or	9
			**Erythroxylaceae**					
Erythroxylum coca Lam. (FC776)	Coca	Cultivated	Blood and cardio-vascular system	Low pressure	Lf	If	Or	2
			Cultural diseases and disorders	Antimonia, gentil, viejo, antiguo	Lf, Sd	Dc, Dd, Fs, If, Ml	Bt, Kn, Or	10
				Susto, espanto	Lf	Dc, Fs, If, Ml	Bt, Or, Pl	11
				Tacsho	Lf	Fs, If	Bt, Or	2
				Pulsario	Lf	If	Or	1
			Dental health	Toothache	Lf	Dc, Fs, f, Ml	Bt, Or, Kn	14
			Digestive system	Stomach cramps	Lf	Dc, If	Or	22
				Diarrhoea	Lf	Fs, If	Or	4
				Stomach pain	Lf	If	Or	2
			Endocrine system	Diabetes	Lf	If	Or	1
			Urinary system	Kidney disorders, emollient, diuretic	Lf	Dd	Or	1
			Infections and infestations	UTA, leishmaniasis	Lf	If	Bt	1
				Malaria	Lf	If	Or	1
			Muscular-skeletal system	Rheumatism	Lf	Ml	Pl	1
			Pregnancy, birth and puerperium	Birth	Lf	Dc, Dd, If	Or	19
				Postpartum	Lf	If	Bt	1
			Reproductive system and reproductive health	Menstruation disorders	Lf	If	Or	2
			**Euphorbiaceae**					
Aleurites moluccanus (L.) Willd.	Nuez de la India	Cultivated	Metabolic system and nutrition	Weight loss	Sd	Ddc	Or	1
Croton perspeciosus Croizat	Sangre de grado	Wild	Other uses	Cancer	Bk, Lx	Fs, Jc	Or	2
			Digestive system	Gastric ulcers	Lx	Fs, If	Or, Pl	16
				Diarrhoea	Lx	Fs, If	Or	5
			Reproductive system and reproductive health	Menstruation disorders	Lx	Ffs	Or, Kn	6
			Skin and subcutaneous tissue	Wounds, healing	Lx	Fs	Bt, Pl	6
				Burns	Lx	Fs	Bt	2
			Urinary system	Prostate disorders	Lx	Fs	Or	1
Euphorbia laurifolia Juss. ex Lam. (FC779)	Lechecaspi, nunumia	Wild	Infections and infestations	Chickenpox	Lx	Fs	Kn	1
				UTA, leishmaniasis	Ep	Ml	Bt	1
			Pregnancy, birth and puerperium	Breastfeeding	Lf, Lx	Fs	Pl, Wh	4
Euphorbia umbellata (Pax) Bruyns	Planta de la vida	Cultivated	Other uses	Cancer	Lx	If	Or	1
			Digestive system	Hepatitis	Lx	If	Or	1
Euphorbia pulcherrima Willd. ex Klotzsch	Flor de pascua	Cultivated	Cultural diseases and disorders	Antimonia, gentil, viejo, antiguo	Lf	Fs	Bt	1
Jatropha curcas L. (FC780)	Piñón, piñones	Wild	Digestive system	Laxative	Sd	Fs, If, Ml	Or	3
Jatropha macrantha Müll. Arg. (FC777)	Huanarpo macho	Wild	Reproductive system and reproductive health	Aphrodisiac	Ep, Fr, Lf, Ro	Dc, Fm, If	Or	6
				Fertility				
Manihot esculenta Crantz (FC781)	Yuca	Cultivated	Cultural diseases and disorders	Tijte	Lx	Fs	Bt	1
			Digestive system	Intestinal parasites	Lf	Dc	Or	1
				Diarrhoea	Ro	Dc, If, Ml	Or	4
Plukenetia volubilis L.	Sacha inchi	Cultivated	Digestive system	Laxative	Sd	Fs	Or	1

Ricinus communis L. (FC778)	Higuerilla	Wild	Digestive system	Stomach cramps	Sd	Oi	Or	1
				Liver disorders	Sd	Oi	Pl	1
				Laxative	Sd	Oi	Or, Pl	13
			Skin and subcutaneous tissue	Burns	Sd	Oi	Pl	1
**Gentianaceae**								
Macrocarpaea revoluta Gilg (FC782)	Tola	Wild	Cultural diseases and disorders	Tijte	Lx	Fs	Pl	1
			Infections and infestations	UTA, leishmaniasis	Sd	Fs	Pl	1
			Digestive system	Diarrhoea	Fr	Fs	Or	1
**Geraniaceae**								
Geranium stuebelii Hieron. (FC783)	Andacushma, tibshilla	Wild	Infections and infestations	UTA, leishmaniasis	Lf	If	Pl	1
Pelargonium roseum Willd. (FC784)	Geranio	Cultivated	Respiratory system	Cough	Lf	If	Gg	1
			Digestive system	Diarrhoea	Lf	If	Or	1
			Skin and subcutaneous tissue	Feet fungus	Lf	Ml	Pl	1
**Hypericaceae**								
Hypericum laricifolium Juss. (FC785)	Chinchango	Wild	Cultural diseases and disorders	Tijte	Ep	If	Bt	1
**Juglandaceae**								
Juglans neotropica Diels (FC786)	Nogal	Wild	Respiratory system	Flu	Lf	If	Or	1
			Urinary system	Prostate disorders	Lf	If	Or	1
			Other uses	Hair loss	Bd, Fr, Lf	Dc, Fm, Fs, If	Bt, Kn	11
			Pregnancy, birth and puerperium	Postpartum	Lf	Dc	Or	1
				Abortive	Lf	If	Bt	1
			Endocrine system	Diabetes	Lf	If	Or	3
**Lamiaceae**								
Clinopodium sericeum (C. Presl ex Benth) Govaerts (FC793)	Romero de monte, romerillo	Wild	Other uses	Hair loss	Ep	If	Bt	1
Lepechinia meyenii (Walp.) Epling (FC795)	Salvia blanca	Wild	Respiratory system	Flu	Fl, Lf	Dc, If	Or	3
			Nervous system and mental health	Insomnia	Lf	If	Or	1
Melissa officinalis L. (FC797)	Toronjil	Cultivated	Blood and cardio-vascular system	High pressure	Ep, Lf	If	Or	5
			Cultural diseases and disorders	Susto, espanto	Lf	If	Or	1
			Digestive system	Stomach cramps	Lf	If	Or	2
			Nervous system and mental health	Sadness	Lf	If	Or	10
			Pregnancy, birth and puerperium	Birth	Lf	If	Or	7
			Reproductive system and reproductive health	Menopause	Lf	If	Or	1
Mentha piperita L. (FC789)	Menta	Cultivated	Blood and cardio-vascular system	Low pressure	Lf	If	Or	2
			Dental health	Toothache	Lf	Fs	Or	2
			Digestive system	Intestinal parasites	Lf	Fs	Or	1
				Stomach cramps	Lf	If	Or	18
				Stomach pain	Lf	If	Or	1
				Diarrhoea	Lf	If	Or	1
			Infections and infestations	Insect bite	Lf	Fs	Kn	1
			Nervous system and mental health	Insomnia	Lf	If	Or	1
			Pregnancy, birth and puerperium	Birth	Lf	If	Or	2
			Reproductive system and reproductive health	Menstruation disorders	Lf	If	Or	5
				Menopause	Lf	If	Or	1
Mentha spicata L. (FC788)	Hierbabuena	Cultivated	Respiratory system	Flu	Lf	Dc, If	Or	6
			Dental health	Toothache	Lf	Dc	Gg	1

			Digestive system	Intestinal parasites	Ep, Lf	Dc, Fs, If, Ml	Or, Vp	90
				Stomach cramps	Lf	If	Or	2
				Diarrhoea	Lf	If	Or	1
				Stomach infection	Lf	If	Or	1
				Laxative	Lf	Dc, If	Or	2
			General ailments with unspecific symptoms	Fever	Lf	If	Or	1
				Headache	Lf	Dc	Or	1
			Infections and infestations	Insect bite	Ep, Lf	If, Ml	Kn, Or, Pl	4
			Reproductive system and reproductive health	Menstruation disorders	Lf	Ml	Kn	1
			Skin and subcutaneous tissue	Wounds, healing	Lf	Ml	Pl	1
				Swelling	Lf	Dc	Or	1
			Dental health	Toothache	Lf	Ml	Or	1
			Cultural diseases and disorders	Tacsho	Ep, Lf	Dc, Fs, If, Ml	Bt, Kn, Or, Vp	218
				Susto, espanto	Lf	Dc, If	Bt, Or, Pl	25
				Aire, malaire	Ep, Lf	Fs, If, Ml	Bt, Or	18
				Antimonia, gentil, viejo, antiguo	Lf	Fs	Bt	1
Minthostachys mollis (Benth.) Griseb. (FC791)	Poleo, ayamanchana, muña	Wild	Infections and infestations	Lice	Ep	Ml	Bt	1
				Insect bite	Lf	Fs, Ml	Kn, Pl	2
				Fleas	Lf	Ml	Or	1
			Blood and cardio-vascular system	Hemorrhoids	Lf	If	Or	1
			Other uses	Cancer	Lf	If	Or	2
			Ritual and magic uses	Curse	Lf	If	Bt	1
			Muscular-skeletal system	Rheumatism	Lf	Fm, Fs, If, Ml, Wm	Bt, Kn	4
			Respiratory system	Flu	Lf	If	Or	4
			Urinary system	Prostate disorders	Lf	If	Or	1
			Reproductive system and reproductive health	Menstruation disorders	Lf	If	Or	1
			Nervous system and mental health	Insomnia	Lf	If	Or	1
			Endocrine system	Diabetes	Lf	Dc, If	Or	2
			Cultural diseases and disorders	Susto, espanto	Lf	If	Or	1
Ocimum basilicum L. (FC787)	Albahaca	Cultivated	Blood and cardio-vascular system	Anemia	Fr, Lf	Fs, Ml	Or	3
			Reproductive system and reproductive health	Menstruation disorders	Lf	If	Or	2
			Ritual and magic uses	Negative vibes	Lf	If	Bt	1
			Pregnancy, birth and puerperium	Birth	Lf	If	Or	6
				Abortive	Ep	If	Or	1
Origanum vulgare L. (FC790)	Orégano	Cultivated	Digestive system	Intestinal parasites	Lf	Fs, If	Or	2
				Stomach cramps	Lf	If	Or	3
			Reproductive system and reproductive health	Menstruation disorders	Ep, Lf	Dc, If	Bt, Kn, Or	40
			Digestive system	Stomach cramps	Ep, Lf	If	Or	100
				Stomach pain	Lf	If	Or	8
			Pregnancy, birth and puerperium	Abortive	Ep, Lf	Dc, Fm, If, Ml	Or	6
				Birth	Lf, Ro	If	Or	6
Rosmarinus officinalis L. (FC792)	Romero de Castilla	Cultivated	Cultural diseases and disorders	Aire, malaire	Eep, Lf	Fm, If	Bt, Kn, Vp	3
				Susto, espanto	Lf	Fm	Bt	1
				Tacsho	Lf	Fm	Pl	1

			Blood and cardio-vascular system	Hemorrhoids	Lf	Fm	Pl	1
			Digestive system	Stomach cramps	Lf	Fm	Or	1
			Ritual and magic uses	Negative vibes	Lf	Fm	Bt	1
			Muscular-skeletal system	Rheumatism	Lf	Fm	Bt	1
			Other uses	Hair loss	Ep, Lf	Fm, If	Bt	9
			Reproductive system and reproductive health	Fertility	Fr	Fm	Or	1
			Skin and subcutaneous tissue	Feet fungus	Lf	Fm	Bt	1
Salvia hispanica L. (FC432)	Chía	Cultivated	Metabolic system and nutrition	Weight loss	Sd	Dc, If, Jc	Or	5
			Other uses	Cancer	Sd	Jc	Or	1
			Blood and cardio-vascular system	Hemorrhoids	Sd	Dc	Or	1
Salvia macrophylla Benth. (FC794)	Salvia azul	Wild	Digestive system	Diarrhoea	Lf	If	Or	1
Stachys arvensis (L.) L. (FC796)	Subsacha	Cultivated	Digestive system	Stomach cramps	Ep, Lf	Dc, If	Or	28
				Diarrhoea	Ep, Lf	Dc, If	Or	15
				Stomach pain	Lf	If	Or	1
				Stomach infection	Lf	If	Or	2
				Laxative	Lf	If	Or	1
			Pregnancy, birth and puerperium	Birth	Lf	If	Or	2
			Skin and subcutaneous tissue	Wounds, healing	Lf	If	Or	1
			Reproductive system and reproductive health	Menstruation disorders	Lf	If	Or	1
Thymus sp.	Tomillo	Cultivated	Digestive system	Diarrhoea	Lf	If	Or	1
				Stomach cramps	Lf	If	Or	1
			**Lauraceae**					
Cinnamomum verum J. Presl (FC798)	Canela	Cultivated	Dental health	Toothache	Bk	Ml	Pl	2
			Pregnancy, birth and puerperium	Abortive	Bk	If	Or	2
			Metabolic system and nutrition	Weight loss	Bk	Dc, If	Or	2
			Blood and cardio-vascular system	Low pressure	Bk	If	Or	1
			Reproductive system and reproductive health	Menstruation disorders	Bk	If	Or	2
			Digestive system	Diarrhoea	Bk	If	Or	1
Nectandra discolor (Kunth) Nees (FC799)	Ishpingo, roble	Wild	Respiratory system	Flu	Lf	If	Or	1
			Cultural diseases and disorders	Antimonia, gentil, viejo, antiguo	Lf	Dc	Or	1
Persea americana Mill. (FC801)	Palta	Cultivated	Cultural diseases and disorders	Aire, malaire	Lf	If	Kn	1
			Digestive system	Diarrhoea	Sd	Dc, If	Or	7
				Stomach cramps	Sd	If	Or	1
				Liver disorders	Sd	If	Or	1
			Other uses	Hair loss	Fr, Sd	Rt	Bt, Kn, Pl	14
				Cancer	Sd	If	Or	1
			Dental health	Toothache	Lf	Fs	Bt	1
			Muscular-skeletal system	Joint sprains	Lf	Fs	Pl	1
			Blood and cardio-vascular system	Hemorrhoids	Sd	If	Or	2
			Pregnancy, birth and puerperium	Abortive	Sd	If	Or	1
			Reproductive system and reproductive health	Aphrodisiac	Fr	Fs	Or	1
			Sensory system	Visual disorders	Lf	If	Pl	1
			Skin and subcutaneous tissue	Feet fungus	Sd	Dc	Or	1

			Urinary system	Kidney disorders, emollient, diuretic	Sd	If	Or	1
Persea subcordata (Ruiz & Pav.) Nees (FC800)	Junjul, junjulí, paccacuna	Wild	Muscular-skeletal system	Joint sprains	Bk, Lf	Fs, Ml, Wm	Bt, Kn, Pl	12
				Rheumatism	Bk	Ml	Pl	1
			**Leguminosae**					
Arachis hypogaea L. (FC812)	Maní	Cultivated	Reproductive system and reproductive health	Aphrodisiac	Fr	Fs	Or	1
Caesalpinia spinosa (Molina) Kuntze (FC818)	Tara, taya	Wild	Cultural diseases and disorders	Tijte	Lx	Fs	Bt	1
			Infections and infestations	UTA, leishmaniasis	Fr	Dc, Dd, Ml	Bt, Kn, Pl	4
			Digestive system	Diarrhoea	Fr	If	Or	1
			Dental health	Toothache	Ffr	Dc	Gg	1
			Skin and subcutaneous tissue	Feet fungus	Fr	Dc, Ml	Bt, Kn	3
				Wounds, healing	Fr	Ml	Kn	1
			Respiratory system	Tonsillitis	Fr, Lf, Sd	If	Gg, Or	38
				Cold	Lf	If	Gg	1
				Flu	Sd	Dc	Or	1
Campsiandra angustifolia Benth.	Huacapurana	Cultivated	Digestive system	Intestinal parasites	Lf	If	Or	1
Cicer arietinum L.	Garbanzo	Cultivated	Blood and cardio-vascular system	Anemia	Fr	Dc	Or	1
Copaifera langsdorffii Desf.	Copaiba	Cultivated, Wild	Other uses	Cancer	Fr	Oi	Or	1
			Digestive system	Diarrhoea	Bk	Oi	Or	1
Crotalaria retusa L.	Espanta muertos	Wild	Cultural diseases and disorders	Tacsho	Lf	Fs	Bt	1
Desmodium molliculum (Kunth) DC. (FC815)	Pie de perro	Wild	Reproductive system and reproductive health	Menstruation disorders	Lf	Dc	Or	1
			Digestive system	Gastric ulcers	Lf	Ml	Or	1
			General ailments with unspecific symptoms	General malaise	Lf	If	Or	1
			Other uses	Cancer	Ep	Dc	Or	1
			Skin and subcutaneous tissue	Wounds, healing	Lf	Dc	Bt	1
			Urinary system	Kidney disorders, emollient, diuretic	Ep, Lf	Dc, If	Or	65
Erythrina edulis Micheli (FC814)	Pajuro	Cultivated	Blood and cardio-vascular system	Anemia	Fr	Dc	Or	1
			Pregnancy, birth and puerperium	Birth	Lf	If	Or	1
				Postpartum	Lf	If	Bt	1
			Cultural diseases and disorders	Pulsario	Bd, Lf	Fs	Bt	2
				Aire, malaire	Lf	Fs	Bt	1
Glycine max (L.) Merr. (FC558)	Soya	Cultivated	Pregnancy, birth and puerperium	Breastfeeding	Fr	Dc, Jc	Or	2
				Birth	Fr	Dc	Or	1
			Reproductive system and reproductive health	Menopause	Fr	Dc, Jc, Ml	Or	10
Glycyrrhiza glabra L.	Regaliz	Wild	Skin and subcutaneous tissue	Feet fungus	Ep	Ml	Kn	
Inga ingoides (Rich.) Willd. (FC809)	Guaba	Wild	Endocrine system	Diabetes	Fr	Fs	Or	2
Lens culinaris Medik.	Lenteja	Cultivated	Cultural diseases and disorders	Susto, espanto	Lf	Dc	Or	1
			Blood and cardio-vascular system	Anemia	Fr	Dc	Or	3
Lupinus exochus C.P. Sm. (FC806)	Chocho de antimonía, chocho del abuelo	Wild	Cultural diseases and disorders	Antimonia, gentil, viejo, antiguo	Fr, Lf	Dc, If	Bt	2
				Susto, espanto	Fr	If	Bt	1
Lupinus mutabilis Sweet. (FC805)	Chocho	Cultivated	Infections and infestations	Chickenpox	Fr	Dc, Ml	Bt	2
				Chicken pest	Fr	Dc	Or	1

				Fleas	Fr	Dc	Bt	1
			General ailments with unspecific symptoms	Fever	Sd	Ml	Pl	1
Medicago sativa L. (FC802)	Alfalfa	Cultivated	Blood and cardio-vascular system	Anemia	Ep, Fr, Lf	If, Jc, Ml	Or	40
			Respiratory system	Flu	Lf	Ml	Or	1
			Reproductive system and reproductive health	Menstruation disorders	Lf, Rro	Dc, If, Ml	Or	6
			Urinary system	Prostate disorders	Lf	Ml	Or	1
			Pregnancy, birth and puerperium	Breastfeeding	Lf	If	Or	2
Ormosia coccinea (Aubl.)Jacks.	Huayruro	Wild	Ritual and magic uses	Bring good luck	Sd	Fs	Nn	2
				Negative vibes	Sd	Fs	Nn	1
Otholobium mexicanum (L.f.) J.W. Grimes (FC807)	Culén chico	Wild	Cultural diseases and disorders	Tacsho	Lf	Fs	Bt	1
			Digestive system	Stomach cramps	Lf	If	Or	3
				Diarrhoea	Lf	If	Or	13
			General ailments with unspecific symptoms	Fever	Lf	If	Or	1
Phaseolus lunatus L.	Pallar	Cultivated	Pregnancy, birth and puerperium	Birth	Lf	If	Or	1
			Skin and subcutaneous tissue	Burns	Lf	Fs	Kn	1
			General ailments with unspecific symptoms	Fever	Lf	If	Bt	1
Phaseolus pachyrrhizoides Harms (FC810)	Habilla	Cultivated	Digestive system	Laxative	Lf	Ml	Or	1
			Sensory system	Hearing disorders	Lf	Ml	Bt	1
				Visual disorders	Lf	Ml	Ew	1
Phaseolus vulgaris L. (FC808)	Frejol	Cultivated	Skin and subcutaneous tissue	Burns	Lf	Fs, Ml	Kn, Pl	2
			Pregnancy, birth and puerperium	Birth	Bd, Lf	If	Or	1
			Infections and infestations	Chickenpox	Fr	Ml	Pl	1
			Blood and cardio-vascular system	Anemia	Fr	Dc	Or	2
			Endocrine system	Diabetes	Bk, Fr	Dc	Or	3
Pisum sativum L. (FC803)	Arveja	Cultivated	Muscular-skeletal system	Joint sprains	Bk	Dc	Pl	1
			Infections and infestations	Chickenpox	Fr	Dc, Ml	Bt, Pl	2
Prosopis pallida (Willd.) Kunth (FC811)	Huarango, algarrobo	Wild	Other uses	Hair loss	St	Dc	Bt	1
Robinia pseudoacacia L. (no voucher specimen)	Flor blanca	Cultivated	Cultural diseases and disorders	Susto, espanto	Lf	Dc	Bt	1
			Infections and infestations	Chickenpox	Fl	Fs	Bt	1
			Blood and cardio-vascular system	High pressure	Fl	Ml	Or	1
			Reproductive system and reproductive health	Menopause	Fl	If	Or	4
Senna bicapsularis (L.) Roxb. (FC364)	Sen, mutuy	Wild	Digestive system	Laxative	Lf	Dc, If	Or	12
				Intestinal parasites	Lf	If	Or	2
Senna multiglandulosa (Jacq.) H.S. Irwin & Barneby (FC813)	Mutuy, muteo Wild	Wild	Cultural diseases and disorders	Susto, espanto	Bd, Lf	If	Bt, Or	2
Spartium junceum L. (FC816)	Retama	Wild	Digestive system	Hepatitis	Fl, Lf	Dc, If	Bt, Or	50
			Infections and infestations	Chickenpox	Fl, Lf	Dc, If	Bt, Or	2
				Malaria	Ep	Dc	Bt	1
Tamarindus indica L. (FC817)	Tamarindo	Cultivated	Digestive system	Laxative	Fr	Jc	Or	1
				Intestinal parasites	Ffr	Jc	Or	1
			Endocrine system	Diabetes	Fr	Ml	Or	1
Tephrosia sinapou (Buc`hoz) A. Chev. (FC804)	Bardasco	Wild	Infections and infestations	Lice	Lf	Ml	Kn	1
Trifolium repens L. (FC819)	Trébol	Wild	Ritual and magic uses	Bring good luck	Lf	Fs	Nn	1
			**Liliaceae**					

Lilium longiflorum Thunb. (FC820)	Azucena, azucena Cultivated	Cultivated	General ailments with unspecific symptoms	Headache	Fr	Fs	Kn	1
			Nervous system and mental health	Sadness	Fl	If	Or	2
			Reproductive system and reproductive health	Fertility	Fr	If	Or	1
			**Linaceae**					
Linum usitatissimum L. (FC821)	Linaza	Cultivated	Metabolic system and nutrition	Weight loss	Sd	Dc, If	Or	2
			Digestive system	Diarrhoea	Sd	Dc, Jc	Or	3
			Pregnancy, birth and puerperium	Birth	Sd	Dc	Or	3
			Urinary system	Kidney disorders, emollient, diuretic	Ssd	Dc, If	Or	22
			**Loasaceae**					
Nasa cuatrecasasii Weigend (FC822)	Ortiga blanca	Wild	Reproductive system and reproductive health	Menstruation disorders	Sd	Ml	Or	1
			**Lythraceae**					
Cuphea ciliata Ruiz & Pav. (FC823)	Chinchimal	Wild	Urinary system	Kidney disorders, emollient, diuretic	Lf	Dc	Or	1
Punica granatum L. (FC824)	Granada	Cultivated	Digestive system	Diarrhoea	Lf	Dc	Or	1
			**Malpighiaceae**					
Bunchosia armeniaca (Cav.) DC.	Cansaboca	Cultivated	Pregnancy, birth and puerperium	Postpartum	Lf	Dc	Or	1
			**Malvaceae**					
Fuertesimalva leptocalyx (Krapov.) Fryxell (FC831)	Malva Wild	Wild	Infections and infestations	Malaria	Lf	If	Or	1
Gossypium hirsutum L. (FC825)	Algodón	Cultivated	Infections and infestations	UTA, leishmaniasis	Sd	Wm	Pl	1
			Sensory system	Hearing disorders	Sd	Fs	Bt	1
			Cultural diseases and disorders	Aire, malaire	Sd	Fs	Bt, Pl	3
				Antimonia, gentil, viejo, antiguo	Sd	Fs	Bt	1
				Tacsho	Sd	If	Or	1
Heliocarpus americanus L. (FC832)	Yansabalsa, yansakiri	Wild	Pregnancy, birth and puerperium	Birth	Bk	If	Or	1
			Digestive system	Diarrhoea	Bk	If	Or	1
Malva arborea (L.) Webb & Berthel. (FC830)	Malva Cultivated	Cultivated	Blood and cardio-vascular system	Hemorrhoids	Lf	Dc	Vp	1
			Reproductive system and reproductive health	Menstruation disorders	Lf	If	Or	1
			Digestive system	Stomach cramps	Lf	If	Or	1
				Diarrhoea	Lf	If	Or	1
				Laxative	Lf	Dc, If	Or	6
			General ailments with unspecific symptoms	Fever	Lf	Dc	Bt	1
			Sensory system	Visual disorders	Lf	Dc, If	Bt	2
			Urinary system	Prostate disorders	Lf	If	Or	1
				Kidney disorders, emollient, diuretic	Ep, Lf	Dc, If	Or	2
			Skin and subcutaneous tissue	Wounds, healing	Lf	Dc	Bt	3
Malvastrum tomentosum (L.) S.R. Hill (FC826)	Angusacha, ancosacha, lancosacha, yator	Wild	Pregnancy, birth and puerperium	Postpartum	Lf	If	Bt	1
			Skin and subcutaneous tissue	Wounds, healing	Lf	Dc, If	Bt, Or	2
			Digestive system	Hepatitis	Lf	Dc	Or	1

			Cultural diseases and disorders	Tijte	Sp	Fs	Wh	1
				Pulsario	Lf	Fs	Pl	1
			Other uses	Cancer	Ep	If	Or	1
				Hair loss	Ep, Lf	Dc	Bt	4
			Muscular-skeletal system	Hernia	Bk	Dc	Pl	1
			Respiratory system	Flu	Lf	If	Or	1
			Sensory system	Visual disorders	Lf	If	Pl	1
			Urinary system	Kidney disorders, emollient, diuretic	Lf	If	Or	1
Ochroma pyramidale (Cav. ex Lam.) Urb. (FC827)	Balsa, palma de mano, palo balsa	Wild	Urinary system	Kidney disorders, emollient, diuretic	Bk	Dc	Or	1
Theobroma cacao L. (FC829)	Cacao	Cultivated	Pregnancy, birth and puerperium	Breastfeeding	Fr	Ml	Or	5
				Birth	Fr	Dc, Ml	Or	6
Triumfetta semitriloba Jacq. (FC828)	Cabayusa	Wild	Infections and infestations	Malaria	Fr	Fm	Kn	1
**Melastomataceae**								
Miconia sp. (FC833)	Shambo	Wild	Infections and infestations	Chickenpox	Lf	If	Bt	1
**Monimiaceae**								
Peumus boldus Molina (FC834)	Boldo	Wild	Nervous system and mental health	Insomnia	Lf	If	Or	1
			Digestive system	Diarrhoea	Lf	Dc	Or	1
**Moraceae**								
Ficus carica L. (FC835)	Higos, higo	Cultivated	Sensory system	Visual disorders	Lf	If	Pl	1
			Cultural diseases and disorders	Tijte	Lx	Fs	Pl	1
				Pulsario	Lf	Dc	Or	1
			Skin and subcutaneous tissue	Burns	Lx	Fs	Pl	1
				Feet fungus	Lx	Fs	Bt	1
			Pregnancy, birth and puerperium	Birth	Lf	If	Or	1
				Breastfeeding	Fr, Lf, Lx	Fs, If	Or, Pl	3
			Other uses	Hair loss	Lf	If	Or	1
Ficus insipida Willd. (FC836)	Higuerón, ojé	Wild	Infections and infestations	UTA, leishmaniasis	Lf	Dc	Bt	1
			Muscular-skeletal system	Joint sprains	Lf	Ml	Pl	1
Ficus maxima Mill. (FC837)	Morero	Wild	Cultural diseases and disorders	Tijte	Ep	Fs	Kn	1
			Dental health	Toothache	Lx, Sd	Fs, Ml	Bt, Kn	2
**Moringaceae**								
Moringa oleifera Lam.	Moringa	Cultivated, wild	Other uses	Cancer	Lf	If	Or	1
			Urinary system	Kidney disorders, emollient, diuretic	Lf	If	Or	1
**Musaceae**								
Musa acuminata Colla (FC839)	Plátano	Cultivated	Blood and cardio-vascular system	Anemia	Fr, Lx, St	If, Ml	Or	2
			Cultural diseases and disorders	Tijte	Bk, Lx	Dc, Fs, Wm	Bt, Kn, Pl	6
			Skin and subcutaneous tissue	Feet fungus	Bk	Dc	Bt, Or	2
			Digestive system	Diarrhoea	Bk, Fr, Lf, Lx	Dc, Fs, If, Ml, Wm	Or	7
				Stomach pain	Fr	Fs	Or	3
				Indigestive	Fr	Fs	Or	20
				Laxative	Fr	Ml	Or	1
				Gastric ulcers	Lx	If, Wm	Or	2
			General ailments with unspecific symptoms	Headache	Fr	Fs	Or	5
			Infections and infestations	Insect bite	Bk	If	Bt	1
				UTA, leishmaniasis	St	Fs	Pl	1

			Muscular-skeletal system	Joint sprains	Bk	Dc	Pl	1
				Muscle cramps	Fr	Fs	Or	1
			Reproductive system and reproductive health	Menstruation disorders	Lx	Fs	Bt	1
			Urinary system	Kidney disorders, emollient, diuretic	Ffr, Lf	Fs	Or, Pl	2
			Pregnancy, birth and puerperium	Breastfeeding	Fr	Fs, If, Ml	Or	5
			Metabolic system and nutrition	Weight loss	Fr	Fs	Or	2
			Skin and subcutaneous tissue	Itil	Bk	Fs	Bt, Pl, Wh	4
				Wounds, healing	Lx	Fs	Bt	1
				Acne	Bk	Fs	Or	1
				Feet fungus	Bk	Dc	Bt	1
Musa x paradisiaca L. (FC838)	Guineo	Cultivated	Cultural diseases and disorders	Tijte	Lx	Fs	Pl	1
			Infections and infestations	Insect bite	Lf	Ml	Kn	1
			Skin and subcutaneous tissue	Itil	Bk	Fs	Pl	1
			Blood and cardio-vascular system	Hemorrhoids	Lx	Fs	Bt	1
			Digestive system	Diarrhoea	St	Fs	Or	1
				Hepatitis	St	Ml	Or	1
		**Myricaceae**						
Morella pubescens (Humb. & Bonpl. ex Willd.) Wilbur (FC840)	Laurel	Wild	Cultural diseases and disorders	Susto, espanto	Lf	Ml	Pl	1
			Infections and infestations	UTA, leishmaniasis	Lf	Dc, Fs, If	Bt, Or, Pl	8
		**Myrtaceae**						
Eucalyptus globulus Labill. (FC842)	Eucalipto	Cultivated	Nervous system and mental health	Insomnia	Lf	If	Or	1
			Respiratory system	Flu	Lf	Dc, If	Bt, Or, Vp	49
				Cough	Bd, Lf	Dc, If, Wm	Or, Pl	5
			General ailments with unspecific symptoms	Fever	Lf	Dc, If	Or	2
				General malaise	Lf	Dc	Bt	1
			Infections and infestations	Insect bite	Lf	Fs, Ml, Wm	Bt, Kn, Or, Pl, Vp	10
Myrcianthes fragans (Sw.) McVaugh (FC844)	Ushún, ushungo	Wild	Muscular-skeletal system	Rheumatism	Lf	Dc, Fm, If, Ml	Bt, Kn	7
				Joint sprains	Lf	Fm, Fs	Kn, Pl	2
			Cultural diseases and disorders	Tijte	Lx	Fs	Bt	1
			Pregnancy, birth and puerperium	Birth	Lf	Fm, If	Or	6
Psidium guajava L. (FC843)	Guayaba	Cultivated	Blood and cardio-vascular system	Anemia	Fr	Dc, Fs	Or	2
			Digestive system	Diarrhoea	Bd, Bk, Fr, Lf	Dc, Fs, If	Or	13
				Laxative	Sd	Ml	Or	1
			Muscular-skeletal system	Bones hardening	Fr	Fs	Or	1
			Other uses	Hair loss	Lf	Dc	Bt	2
			Pregnancy, birth and puerperium	Birth	Lf	If	Or	1
			Nervous system and mental health	Mental stimulant	Fr	Fs	Or	1
			Urinary system	Prostate disorders	Lf	If	Or	1
Syzygium aromaticum (L.) Merr. & L.M. Perry (FC841)	Clavo de olor	Cultivated	Digestive system	Diarrhoea	Fl	If	Or	1
			Dental health	Cavity	Fl	Fs, Ml	Bt, Or, Pl	8

				Toothache	Fl, Fr, Sd, St	Fs, Ml	Bt, Or, Pl	61
			**Oleaceae**					
Olea europaea L. (FC845)	Olivo	Cultivated	Other uses	Hair loss	Fr	Oi	Bt	1
			Digestive system	Laxative	Fr	Oi	Or	1
			**Onagraceae**					
Fuchsia rivularis J.F. Macbr.	Santa Lucía	Wild	Skin and subcutaneous tissue	Wounds, healing	Lf	Fs	Kn	1
Oenothera rosea L`Hér. ex Aiton (FC846)	Chupasangre	Wild	Reproductive system and reproductive health	Menstruation disorders	Ep	Ml	Kn	1
			**Orchidaceae**					
Epidendrum laciniitropis Hágsater (FC848)	Monterrosa	Wild	Cultural diseases and disorders	Pulsario	Fl	If	Or	1
Epidendrum secundum Jacq. (FC847)	Inguilito, inguil	Wild	Nervous system and mental health	Sadness	Fl	Dc	Or	1
			**Oxalidaceae**					
Averrhoa carambola L. (FC849)	Carambola	Cultivated	Blood and cardio-vascular system	High pressure	Fr	Jc, Mml	Or	5
			Pregnancy, birth and puerperium	Birth	Fr	Jc	Or	1
Oxalis tuberosa Molina (FC850)	Ocas	Cultivated	Other uses	Cancer	Ro	Ml	Or	1
			Urinary system	Prostate disorders	Ro	Ml	Or	1
			**Papaveraceae**					
Bocconia integrifolia Bonpl. (FC851)	Huacango, atujtallga, mangapaqui, mangapaque, sandoval	Wild	Cultural diseases and disorders	Antimonia, gentil, viejo, antiguo	Lf	If	Bt	1
			**Passifloraceae**					
Passiflora edulis Sims. (FC853)	Maracuyá	Cultivated	Blood and cardio-vascular system	High pressure	Fr, Sd	Fs, If, Jc, Ml	Or	46
			General ailments with unspecific symptoms	Headache	Fr	If	Or	1
			Digestive system	Intestinal parasites	Fr	Jc	Or	1
				Laxative	Fr	Jc	Or	1
			Reproductive system and reproductive health	Menopause	Ffr, Lf	Jc, Ml	Or	2
Passiflora ligularis Juss. (FC852)	Granadilla	Cultivated	Respiratory system	Cold	Fr	Fs	Or	1
			General ailments with unspecific symptoms	Fever	Lf	If	Or	1
			Digestive system	Indigestive	Fr	Fs	Or	1
				Constipation	Fr	Fs	Or	4
				Intestinal infection	Fr	Fs	Or	1
				Liver disorders	Fr	Fs	Or	1
				Laxative	Fr, Lf, Sd	Fs, If, Jc	Or	15
			Urinary system	Prostate disorders	Fr	Fs	Or	1
				Kidney disorders, emollient, diuretic	Fr, Lf	Fs, If, Wm	Or, Pl	6
			Cultural diseases and disorders	Pulsario	Lf	Fs	Pl	1
				Susto, espanto	Lf, Sd	Dc, If	Bt, Or	3
Passiflora tripartita (Juss.) Poir. var. *mollisima* (Kunth.) Holm-Niels. & P.M. Jørg. (FC854)	Pur pur	Cultivated	Muscular-skeletal system	Rheumatism	Fr	If	Or	1
				Joint sprains	Fr, Lf	Fs, If, Ml, Wm	Or, Pl	5
			Pregnancy, birth and puerperium	Birth	Fr, Lf	Dc, Fs	Or	3
			Urinary system	Kidney disorders, emollient, diuretic	Lf	Dc	Or	1
			Reproductive system and reproductive health	Menstruation disorders	Fr	Dc	Or	1

**Pedaliaceae**								
Sesamum indicum L. (FC855)	Ajonjolí	Wild	Reproductive system and reproductive health	Menopause	Fr	Ml	Or	1
			Digestive system	Diarrhoea	Bk	If	Or	1
			Muscular-skeletal system	Hernia	Bk	Dc	Or	1
**Phyllanthaceae**								
Phyllanthus niruri L. (FC856)	Chancapiedra	Wild	Urinary system	Prostate disorders	Ep, Lf	If	Or	8
				Kidney disorders, emollient, diuretic	Ap, Ep, Lf	Dc, If	Or	38
			Reproductive system and reproductive health	Menstruation disorders	Lf	If	Or	1
			Skin and subcutaneous tissue	Wounds, healing	Ep	If	Or	1
**Phytolaccaceae**								
Phytolacca bogotensis Kunth (FC857)	Airambo	Wild	Cultural diseases and disorders	Susto, espanto	Ep	If	Bt	1
			Infections and infestations	Fleas	Ep, Ssd	Dc, If	Bt, Or	3
**Pinaceae**								
Pinus radiata D. Don (FC858)	Pino	Cultivated	Dental health	Toothache	Lx	Fs	Pl	1
**Piperaceae**								
Peperomia microphylla Kunth (FC859)	Congona Cultivated	Cultivated	Cultural diseases and disorders	Susto, espanto	Lf	If	Bt, Or	2
			Cultural diseases and disorders	Sadness	Lf	If	Or	9
				Insomnia	Ap, Lf	If	Or	2
			Blood and cardio-vascular system	High pressure	Lf	If	Or	1
			Digestive system	Stomach cramps	Lf	If	Or	2
			Reproductive system and reproductive health	Menopause	Lf	If	Or	1
Piper acutifolium Ruiz & Pav. (FC860)	Matico de huerta	Cultivated	Infections and infestations	UTA, leishmaniasis	Lf	Dc	Bt	1
				Chickenpox	Lf	If	Bt	1
			Muscular-skeletal system	Joint sprains	Lf	Fs	Pl	1
			Other uses	Cancer	Lf	If	Or	2
				Hair loss	Lf	Dc	Bt	1
			Reproductive system and reproductive health	Vaginal infection	Lf	If	Or	1
			Skin and subcutaneous tissue	Acne	Lf	Ml	Pl	1
				Wounds, healing	Lf	Dc, If, Ml	Bt, Kn, Or	10
				Burns	Lf	Dd, Ml	Bt, Kn	3
			Digestive system	Stomach cramps	Lf	Dc, If	Or	5
				Diarrhoea	Lf	If	Or	2
				Gastric ulcers	Lf	Dc, If	Or	2
			Blood and cardio-vascular system	Hemorrhoids	Lf	Dc	Vp	1
				High pressure	Lf	If	Or	1
			Respiratory system	Flu	Lf	If	Bt	46
				Cough	Lf	Dc, If	Or	15
			Urinary system	Prostate disorders	Lf	If	Or	2
				Kidney disorders, emollient, diuretic	Lf	If	Or	3
Piper aequale Vahl.	Cordoncillo pequeño	Wild	Cultural diseases and disorders	Susto, espanto	Lf	If	Bt	1
Piper nigrum L.	Pimienta	Cultivated	Dental health	Toothache	Fr	Fs	Pl	1
**Plantaginaceae**								
Globularia alypum L.	Coronilla	Cultivated	Pregnancy, birth and puerperium	Birth	Lf	If	Or	1
Plantago major L. (FC861)	Llantén	Cultivated	Skin and subcutaneous tissue	Wounds, healing	Lf	Dc, Fs, If, Ml, Wm	Bt, Kn, Or, Pl	93

				Feet fungus	Lf	Dc	Bt	1
				Burns	Lf	Dc, Fs, If, Ml, Wm	Bt, Or, Pl	10
			Other uses	Hair loss	Lf	Dc, If	Bt	2
			Dental health	Toothache	Lf	Dc, If, Wm	Bt, Gg, Pl	5
			Digestive system	Stomach cramps	Lf	If	Or	1
			General ailments with unspecific symptoms	Fever	Lf	Dc, Fs, If	Bt, Or	3
			Infections and infestations	Insect bite	Lf	Dc, Ml	Bt, Kn	3
				Chickenpox	Lf	Dc	Bt	1
			Blood and cardio-vascular system	Hemorrhoids	Lf	Dc, If	Bt, Vp	5
			Pregnancy, birth and puerperium	Postpartum	Lf	If	Bt	1
			Muscular-skeletal system	Joint sprains	Lf	Fs	Kn	1
				Rheumatism	Lf	Fs	Pl	1
			Nervous system and mental health	Insomnia	Lf	If	Or	1
			Reproductive system and reproductive health	Menstruation disorders	Lf	If, Ml	Bt, Or, Pl	4
			Sensory system	Visual disorders	Lf	Dc, If	Bt, Or	6
			Urinary system	Kidney disorders, emollient, diuretic	Lf	Ddc, Fs, If, Ml	Or, Pl	21
				Prostate disorders	Lf	If	Or	1
Avena sativa L.	Avena	Cultivated	Pregnancy, birth and puerperium	Breastfeeding	Sd	Dc, Ml	Or	3
			Skin and subcutaneous tissue	Acne	Sd	Dc	Bt	1
Cortaderia jubata (Lemoine ex Carrière) Stapf. (FC867)	Cortadera	Wild	Reproductive system and reproductive health	Menopause	Ro	Dc	Or	1
			Pregnancy, birth and puerperium	Birth	Ro	Ddc, Fs, If, Ml	Or	1
Cymbopogon citratus (DC.) Stapf (FC869)	Hierbaluisa	Cultivated	Pregnancy, birth and puerperium	Birth	Lf	If	Or	1
			Digestive system	Stomach cramps	Lf	If	Or	1
				Diarrhoea	Lf	Or	Or	1
				Laxative	Lf	If	Or	1
			General ailments with unspecific symptoms	Fever	Lf	If	Or	1
			Blood and cardio-vascular system	High pressure	Lf	If	Or	2
			Nervous system and mental health	Insomnia	Lf	If	Or	2
			Reproductive system and reproductive health	Menstruation disorders	Lf	If	Or	2
				Menopause	Ep	If	Or	1
			Respiratory system	Flu	Lf	If	Or	1
				Couh	Lf	If	Or	1
Cynodon dactylon (L.) Pers. (FC868)	Grama dulce	Wild	Blood and cardio-vascular system	Hemorrhoids	Lf	Ffs	Bt	1
Gynerium sagittatum (Aubl.) P. Beauv. (FC863)	Caña brava	Wild	Urinary system	Prostate disorders	Lf	If	Or	1
Hordeum vulgare L. (FC866)	Cebada	Cultivated	Metabolic system and nutrition	Weight loss	Sd	Dc	Or	1
			Urinary system	Prostate disorders	Sd	Dc	Or	1
				Kidney disorders, emollient, diuretic	Lf, Sd	Dc, If	Or	28

Oryza sativa L. (FC862)	Arroz	Cultivated	Digestive system	Diarrhoea	Fr	Dc, If, Wm	Or	5
			Other uses	Hair loss	Sd	Fs	Pl	1
Phragmites australis (Cav.) Trin. ex Stend (FC865)	Carricillo, carrizo, timbuche	Wild	Other uses	Hair loss	Bd, Lf, St	Ddc, Fs, If, Ml	Bt, Or	3
			Urinary system	Prostate disorders	Bk	Dc	Or	1
Saccharum officinarum L. (FC864)	Caña de azúcar	Cultivated	Endocrine system	Diabetes	Bk	Dc	Or	1
			Respiratory system	Flu	St	Fm	Pl	1
			Infections and infestations	Insect bite	Fr	Fm	Or	1
				UTA, leishmaniasis	St	Fm	Bt	1
Zea mays L. (FC870)	Maíz	Cultivated	Infections and infestations	Chickenpox	Fr	Dc, Fs, If, Ml	Bt, Kn, Or, Pl	69
			Pregnancy, birth and puerperium	Breastfeeding	Fr	Dc, If, Ml, Wm	Bt, Or	8
			Digestive system	Intestinal parasites	Fr	Ml	Pl	1
				Stomach cramps	Ss	If	Or	1
			Cultural diseases and disorders	Antimonia, gentil, viejo, antiguo	Fr	Ddc, Fs, If, Ml	Bt, Pl	5
				Susto, espanto	Fr	Dd, Ml	Bt, Kn, Or, Pl	6
				Tijte	Ss	Wm	Bt	1
			Blood and cardio-vascular system	High pressure	Fr	Dc, Ml	Or	7
				Anemia	Fr	Ml	Or	1
			Skin and subcutaneous tissue	Burns	Fr	If	Or	1
			Reproductive system and reproductive health	Menopause	Fr	Dc	Or	1
			Urinary system	Kidney disorders, emollient, diuretic	Ss	Ddc, Fs, If, Ml	Or	7
				Prostate disorders	Ss	If	Or	1
			**Polygalaceae**					
Polygala paniculata L. (FC871)	Canchalagua	Wild	Digestive system	Stomach cramps	Lf	If	Or	2
			Skin and subcutaneous tissue	Acne	Lf	Dc	Or	1
			**Polygonaceae**					
Polygonum hydropiperoides Michx. (FC873)	Shiñachirapa	Wild	Cultural diseases and disorders	Antimonia, gentil, viejo, antiguo	Lf	If	Bt	1
Rumex obtusifolius L. (FC872)	Malayerba, hierbamaría	Wild	General ailments with unspecific symptoms	Fever	Lf, Ro	Dc, If	Or	2
			Infections and infestations	Insect bite	Lf	Ml	Kn	1
			Urinary system	Kidney disorders, emollient, diuretic	Lf	If	Or	1
			Digestive system	Intestinal parasites	Ro	Ml	Or	1
				Hepatitis	Lf, Ro	If, Ml	Or	2
				Laxative	Lf, Ro	If	Or	4
Rumex peruanus Rech. f. (FC874)	Canchil, unduluj, undulón	Wild	Skin and subcutaneous tissue	Burns	Ro	Fs	Pl	1
			Other uses	Cancer	St	If	Pl	1
Portulaca oleraceae L.	Verdolaga	Wild	Sensory system	Hearing disorders	Lf	Fs	Pl	1
Oreocallis grandiflora (Lam.) R.Br. (FC875)	Saltaperico, palpal, avincho, rumilanche	Wild	Other uses	Cancer	Fl	Dc	Or	1
			Dental health	Toothache	Sd	Ml	Or	1
			Blood and cardio-vascular system	Hemorrhoids	Fl, Lf	Dc	Or	2
			Digestive system	Stomach cramps	Lf	If	Or	1

			Urinary system	Prostate disorders	Lf	If	Or	2
				Kidney disorders, emollient, diuretic	Fl, Lf	Dc, If	Or	7
**Ranunculaceae**								
Clematis haenkeana C. Presl (FC876)	Pumashaire, sajshauar	Wild	Infections and infestations	UTA, leishmaniasis	Lf	Ml, Wm	Pl	2
**Rosaceae**								
Cydonia oblonga Mill. (FC879)	Membrillo	Cultivated	Digestive system	Diarrhoea	Fr	If	Or	1
			Endocrine system	Diabetes	Fr	Dc	Or	1
Filipendula ulmaria (L.) Maxim.	Reina Isabel	Wild	Infections and infestations	UTA, leishmaniasis	Fl, Lf	Fs, If	Or, Pl	2
Fragaria × ananassa (Weston) Duchesne	Fresa	Wild	Urinary system	Kidney disorders, emollient, diuretic	Fr	Fs	Or	1
Malus domestica Borkh. (FC878)	Manzana serrana, manazanita de huerta	Cultivated	Blood and cardio-vascular system	Anemia	Fr	Ml	Or	5
			Digestive system	Diarrhoea	Fr	Dc, If	Or	3
				Stomach pain	Fr	Fs	Or	1
				Indigestive	Fr	Fs	Or	7
				Bad breath	Fr	Fs	Or	1
			Endocrine system	Diabetes	Fr	Fs	Or	1
			Nervous system and mental health	Mental stimulant	Fr	Fs	Or	2
				Stress	Fr	If	Or	2
				Insomnia	Fr, Lf	Dc, If	Or	96
				Sadness	Fr	Dc	Or	1
			Skin and subcutaneous tissue	Acne	Fr	Fs	Or	1
			Pregnancy, birth and puerperium	Birth	Fr	Dc	Or	1
			Dental health	Toothache	Fr	Fs	Or	2
			Metabolic system and nutrition	Weight loss	Fr	Dc	Or	1
Potentilla anserina L.	Potentila	Wild	Nervous system and mental health	Epilepsy	Eep	Dc	Or	1
Prunus dulcis (Mill.) D.A. Webb	Almendra	Cultivated	Blood and cardio-vascular system	Anemia	Fr	Fs	Or	1
Prunus persica (L.) Batsch (FC877)	Durazno	Cultivated	Cultural diseases and disorders	Aire, malaire	Lf	Dc, Fs, If	Bt, Or	11
				Susto, espanto	Lf	Fs	Bt	2
			Digestive system	Diarrhoea	Lf	If	Or	2
			General ailments with unspecific symptoms	Fever	Lf	Dc, Fs, If	Bt	5
			Nervous system and mental health	Insomnia	Lf	If	Or	1
				Sadness	Lf	If	Or	1
				Mental stimulant	Fr	Fs	Or	2
			Pregnancy, birth and puerperium	Postpartum	Lf	Ddc, If	Bt, Pl	8
				Birth	Lf	If	Bt	1
Prunus serotina Ehrh.	Capulí	Wild	Infections and infestations	UTA, leishmaniasis	Lf	Fs	Pl	1
			Reproductive system and reproductive health	Menopause	St	If	Or	1
Pyrus sp.	Pera	Cultivated	Digestive system	Indigestive	Fr	Fs	Or	1
Rosa centifolia L. (FC881)	Rosa, rosa blanca	Cultivated	Nervous system and mental health	Sadness	Fl	If	Or	1
			Sensory system	Visual disorders	Fl	Fs, Ml	Bt, Kn	3
Rubus megalococcus Focke (FC880)	Mora	Wild	Sensory system	Visual disorders	Fl	If	Bbt, Or	2
			Endocrine system	Diabetes	Lf	If	Or	2
			Reproductive system and reproductive health	Menstruation disorders	Fr	Dc	Or	1
			Respiratory system	Flu	Fl, Lf	Dc, If	Or	3
				Cough	Fl	If	Or	2
**Rubiaceae**								

Cinchona krauseana L. Andersson (FC885)	Tafitán	Wild	Muscular-skeletal system	Joint sprains	Lf	Fs	Pl	1
Cinchona officinalis L. (FC884)	Cascarilla, quina	Wild	Cultural diseases and disorders	Antimonia, gentil, viejo, antiguo	Lf	Fm	Or	1
			Infections and infestations	Malaria	Bk, Lf	Dc, Fm, If	Or	3
Coffea arabica L. (FC882)	Café	Cultivated	Blood and cardio-vascular system	Low pressure	Fr, Sd	Dc, If	Or	34
			Reproductive system and reproductive health	Menstruation disorders	Lf	Dc	Or	1
			Metabolic system and nutrition	Weight loss	Fr	If	Or	1
			Muscular-skeletal system	Rheumatism	Lf	Fm	Kn	1
			Pregnancy, birth and puerperium	Breastfeeding	Bd	If	Or	1
			Respiratory system	Cough	Ffr	If	Or	1
Morinda citrifolia L. (FC883)	Noni	Cultivated	Other uses	Cancer	Fr	Fs, Jc, Ml	Or	5
			Digestive system	Diarrhoea	Fr	Ml	Or	2
			Urinary system	Prostate disorders	Fr	Jc	Or	1
				Kidney disorders, emollient, diuretic	Lf	If	Or	1
Uncaria tomentosa (Willd. ex Schult.) DC. (FC886)	Uña de gato	Wild	Other uses	Cancer	Lf, St	Dc, If	Or	3
			Digestive system	Diarrhoea	Bk	Dc	Or	1
			Reproductive system and reproductive health	Fertility	Ep	Fm	Or	1
				Aphrodisiac	Bk	If	Or	1
			Skin and subcutaneous tissue	Feet fungus	Bk	Dc	Bt	1
			Urinary system	Kidney disorders, emollient, diuretic	Ep, Lf	Dc	Or	3
				Prostate disorders	Bk	Dc, Fm	Or	2
			**Rutaceae**					
Citrus aurantiifolia (Christm.) Swingle (FC887)	Lima	Cultivated	Blood and cardio-vascular system	High pressure	Fl, Ffr	Fs, If, Jc, Ml	Or	23
			Cultural diseases and disorders	Susto, espanto	Ffr	Jc	Or	1
			Digestive system	Hepatitis	Fl, Ffr	Fs, If	Or	2
			General ailments with unspecific symptoms	Fever	Fr, Lf	Dc, Jc	Bt, Or	2
			Respiratory system	Flu	Fr	Fs	Or	1
			Nervous system and mental health	Sadness	Fr	Fs	Or	1
				Mental stimulant	Fr	Fs	Or	1
			Endocrine system	Diabetes	Fr	Fs	Or	1
			Skin and subcutaneous tissue	Burns	Fr	Ml	Bt	1
			Sensory system	Visual disorders	Fr	Fs, Jc, Ml	Bt, Ew, Kn, Or, Pl	26
Citrus aurantium L. var. *amara* L. (FC891)	Naranjo	Cultivated	Pregnancy, birth and puerperium	Birth	Lf	If	Or	1
			Nervous system and mental health	Insomnia	Bd, Lf	If	Or	2
			Reproductive system and reproductive health	Menopause	Lf	If	Or	1
Citrus aurantium L. var. *sinensis* L. (FC890)	Naranja	Cultivated	General ailments with unspecific symptoms	Fever	Lf	Fm	Kn	1
			Pregnancy, birth and puerperium	Birth	Ec, Fr	Fs, If	Or	2

			Digestive system	Intestinal parasites	Fr	Jc	Or	1
				Laxative	Fr, Sd	Dc, Jc, Ml	Or	5
				Diarrhoea	Ffr	Fs, Jc	Or	2
			Blood and cardio-vascular system	High pressure	Fr	Dc, Fs, If	Or	4
				Anemia	Fr	Ml	Or	1
			Respiratory system	Flu	Fr	Dc, Fs, If, Jc	Or	32
			Muscular-skeletal system	Muscle cramps	Fr	Fs	Or	1
			Nervous system and mental health	Insomnia	Fr, Lf	Fs, If, Jc	Or	3
				Mental stimulant	Fr	Fs	Or	3
				Sadness	Lf	If	Or	1
			Skin and subcutaneous tissue	Feet fungus	Fr	Fs, Jc, Ml, Wm	Bt, Kn, Or, Pl	13
				Wounds, healing	Fr	Fs, Jc	Bt	3
				Acne	Fr	Fs, If, Jc	Bt, Or	5
				Burns	Ec, Fr	Fs, If	Bt, Kn, Or, Pl	5
			Endocrine system	Diabetes	Fr	Fs	Or	1
			Nervous system and mental health	Insomnia	Ffr	If	Or	1
Citrus limon (L.) Osbeck (FC888)	Limón, limón ácido	Cultivated	Digestive system	Intestinal parasites	Ssd	Ml	Or	1
				Stomach cramps	Fr	If	Or	1
				Diarrhoea	Fr	If, Jc, Wm	Or	11
				Stomach pain	Fr	If	Or	1
				Hepatitis	Fr	Jc	Or	1
				Laxative	Ssd	Ml	Or	1
			General ailments with unspecific symptoms	Fever	Fr	Dc, Fs, If, Jc, Ml	Bt, Or, Pl	10
				General malaise	Fr	Jc	Or	1
			Infections and infestations	Insect bite	Fr	Fs, Jc, Ml	Bbt, Or, Pl	18
				UTA, leishmaniasis	Fr	Fs, Jc	Bt	2
			Metabolic system and nutrition	Weight loss	Fr	If, Jc	Or	6
			Muscular-skeletal system	Rheumatism	Lf	Fm	Kn	1
			Dental health	Cavity	Sd	Fs	Pl	2
				Toothache	Fr, Sd	Fs, Ml	Bt, Kn, Or, Pl	13
			Pregnancy, birth and puerperium	Postpartum	Fr	If	Or	1
			Reproductive system and reproductive health	Menstruation disorders	Fr	Fs, If	Bt, Or	2
			Respiratory system	Flu	Fr, Lf	Dc, Fs, If, Jc, Wm	Or	59
				Cough	Fr	If, Jc	Gg, Or	4
			Sensory system	Visual disorders	Fr	Fs, Ml	Bt, Kn	2
			Ritual and magic uses	Negative vibes	Fr	Fs	Nn	2
Citrus paradisi Macfad. (FC893)	Toronja	Cultivated	Metabolic system and nutrition	Weight loss	Fr	Jc	Or	2
			Blood and cardio-vascular system	High pressure	Fr	Jc	Or	1
			Reproductive system and reproductive health	Fertility	Fr	If, Jc	Or	1
Citrus reticulata Blanco (FC889)	Mandarina	Cultivated	Respiratory system	Flu	Fr	Fs	Or	2
			Nervous system and mental health	Mental stimulant	Fr	Fs	Or	1
Ruta chalepensis L. (FC892)	Ruda	Cultivated	Dental health	Toothache	Lf	Ml	Kn, Or, Pl	4
			Cultural diseases and disorders	Aire, malaire	Lf	Fs, If	Bt, In, Or	42

				Antimonia, gentil, viejo, antiguo	Lf	Ffs	Bt	1
				Susto, espanto	Lf	Fs, If	Bt, Or	10
				Tacsho	Lf	If	Bt, Or	3
			Digestive system	Intestinal parasites	Lf	Ml	Or	1
				Stomach cramps	Lf	If	Or	4
			Sensory system	Hearing disorders	Lf	Fs, If	Pl	2
			Infections and infestations	Chickenpox	Lf	Dc	Bt	1
			Muscular-skeletal system	Rheumatism	Lf	Fm, If	Bt	2
			Pregnancy, birth and puerperium	Abortive	Ap, Ep, Lf	Dc, Fm, Fs, If, Ml	Or	19
				Postpartum	Lf	If	Bt, Or	2
			General ailments with unspecific symptoms	Fever	Lf	Fm	Kn	1
				Headache	Ep	Fs	Bt	1
			Ritual and magic uses	Curse	Lf	Ml	Bt	1
				Bring good luck	Ap, Ep, Fl, Lf	Ddc, Fs, If, Ml	Bt, Kn, Nn	14
				Negative vibes	Ep, Lf	Fs, If	Bt, Nn	6
**Salicaceae**								
Salix humboldtiana Willd. (FC894)	Saúce, álamo	Wild	Blood and cardio-vascular system	Low pressure	Lf	If	Or	1
			Other uses	Hair loss	Lf	Dc, Fm, Fs, If	Bt	13
			Muscular-skeletal system	Rheumatism	Lf	Dc	Bt	1
**Santalaceae**								
Phoradendron nervosum Oliv. (FC895)	Suelda consuelda	Wild	Muscular-skeletal system	Hernia	Lf	Ml	Pl	1
				Rheumatism	Lf	Fs	Bt	1
				Joint sprains	Lf	Fs, If, Ml, Wm	Bt, Pl	26
**Sapindaceae**								
Dodonaea viscosa (L.) Jacq. (FC896)	Chamana	Wild	Cultural diseases and disorders	Susto, espanto	Lf	If	Bt	2
			Infections and infestations	Chickenpox	Lf	If	Bt	1
			Muscular-skeletal system	Rheumatism	Lf	Dc	Bt	1
				Joint sprains	Lf	Wm	Ppl	1
			Skin and subcutaneous tissue	Burns	Lf	Ml	Pl	1
Sapindus saponaria L. (FC897)	Choloque	Wild	Infections and infestations	Fleas	Sd	Dc	Bt	1
**Sapotaceae**								
Pouteria lucuma (Ruiz & Pav.) Kuntze (FC898)	Lúcuma	Wild	Pregnancy, birth and puerperium	Breastfeeding	Bd, Lf	Dc, If	Or	7
**Scrophulariaceae**								
Alonsoa meridionalis (L. f.) Kuntze (FC899)	Duraznillo	Wild	Infections and infestations	UTA, leishmaniasis	Lx	Fs	Pl	1
Buddleja blattaria J.F.Macbr. (FC900)	Utcusacha, flor blanca	Wild	Pregnancy, birth and puerperium	Bi	Lf	If	Or	1
			Infections and infestations	UTA, leishmaniasis	Lf	Ml	Pl	2
**Siparunaceae**								
Siparuna muricata (Ruiz & Pav.) A. DC. (FC901)	Payón, paigamo, poshmete, añasquero	Wild	Cultural diseases and disorders	Antimonia, gentil, viejo, antiguo	Lf	If	Bt	1
				Susto, espanto	Lf	If	Bt	2
			Pregnancy, birth and puerperium	Postpartum	Lf	If	Bt	1
**Solanaceae**								
Capsicum annuum L.	Pimiento rojo	Cultivated	Digestive system	Stomach cramps	Fr	Wm	Or	1
Capsicum pubescens Ruiz & Pav. (FC903)	Ají, ají rocoto	Cultivated	General ailments with unspecific symptoms	Fever	Lf	If	Bt	2
			Pregnancy, birth and puerperium	Postpartum	Lf	Fs, If, Ml	Bt, Kn, Or	58
			Skin and subcutaneous tissue	Burns	Fr, Lf	Fs, If, Mml	Bt, Kn, Or, Pl	14

				Feet fungus	Fr	Fs	Kn	1
			Cultural diseases and disorders	Susto, espanto	Lf	Dc, If	Bt	2
			Digestive system	Intestinal parasites	Lf	If	Or	1
				Diarrhoea	Lf	Ml	Or	1
			General ailments with unspecific symptoms	Fever	Ep, Lf	Dc, If, Ml	Bt, Or, Pl	32
				General malaise	Lf	If	Bt	1
Cestrum auriculatum L`Hér (FC907)	Hierbasanta	Wild	Pregnancy, birth and puerperium	Abortive	Lf	Fm	Or	1
				Postpartum	Lf	If	Bt	1
			Infections and infestations	Chickenpox	Lf	If	Bt	1
				Lice	Lf	Dc	Bt	1
			Urinary system	Prostate disorders	Lf	If	Bt	1
			Reproductive system and reproductive health	Menstruation disorders	Ro	Dc	Or	2
			Skin and subcutaneous tissue	Wounds, healing	Lf	Dc, If	Bt	2
				Feet fungus	Lf	Dc	Bt	1
Cestrum tomentosum L. f. (FC908)	Huaspasacha de la jalca	Wild	Digestive system	Stomach cramps	Lf	If	Or	2
			Reproductive system and reproductive health	Menstruation disorders	Lf	If	Or	1
			Blood and cardio-vascular system	High pressure	Fr	Fs, Jc, Ml	Or	6
				Hemorrhoids	Fr	Ml	Kn	1
Cyphomandra betacea Cav. (FC913)	Tomate de árbol, pepino de árbol, berenjena de árbol	Cultivated	Endocrine system	Diabetes	Fr	Fs, Ml	Or	4
			Metabolic system and nutrition	Weight loss	Fr	Fs, If, Jc, Mml	Or	13
			Infections and infestations	Malaria	Fr	Fs	Or	1
			Digestive system	Diarrhoea	Fr	Fs	Or	1
			Cultural diseases and disorders	Susto, espanto	Lf	If	Bt	1
Datura stramonium L. (FC904)	Chamico, datura	Wild	Blood and cardio-vascular system	Hemorrhoids	Lf	Ml, Wm	Pl	2
			Ritual and magic uses	Curse	Lf	Dc	Bt	1
			Cultural diseases and disorders	Susto, espanto	Lf	Dd	Bt	1
				Antimonia, gentil, viejo, antiguo	Lf	Dd	Bt	1
			Infections and infestations	UTA, leishmaniasis	lF	Ml	Pl	1
Nicotiana setchellii Goodsp. (FC911)	Tabaco	Cultivated	Blood and cardio-vascular system	Hemorrhoids	Lf	Wm	Bt	1
			Skin and subcutaneous tissue	Swelling	Lf	Ml	Pl	1
			Sensory system	Hearing disorders	Lf	Ml	Pl	1
			Skin and subcutaneous tissue	Burns	Lf	If	Pl	1
			General ailments with unspecific symptoms	Fever	Lf	Dc, Fs, If	Bt	4
				General malaise	Lf	Dc	Bt	1
Physalis peruviana L. (FC902)	Aguaymanto, tomatillo	Wild	Pregnancy, birth and puerperium	Birth	Fr	Jc	Or	1
				Postpartum	Lf	Fs, If	Bt	2
			Digestive system	Diarrhoea	Lf	If	Or	1
			Respiratory system	Flu	Fr	Fm	Or	1
			Endocrine system	Diabetes	Fr	Fs	Or	1
			General ailments with unspecific symptoms	Fever	Lf	If	Bt	2
			Sensory system	Visual disorders	Lf	Dc	Bt	1
Solanum americanum Mill. (FC906)	Hierbamora, cuash	Wild		Hearing disorders	Lf	Fs	Kn	1
			Infections and infestations	Fleas	Lf	Dc	Bt	1
			Skin and subcutaneous tissue	Feet fungus	Sd	If	Or	1
Solanum interandinum Bitter (FC909)	Mushañao	Wild	General ailments with unspecific symptoms	Fever	Lf	Fs, If	Bt	2

			Pregnancy, birth and puerperium	Postpartum	Lf	If	Or	1
			General ailments with unspecific symptoms	Fever	Lf	Fs, If	Bt	2
Solanum lycopersicum L. (FC912)	Tomate	Cultivated	Other uses	Cancer	Fr	Jc	Or	1
			Blood and cardio-vascular system	Anemia	Fr	Ml	Or	1
			Sensory system	Visual disorders	Fr	Fs	Pl	1
			Skin and subcutaneous tissue	Burns	Fr, Lf	Fs, If	Bt, Kn, Or, Pl	71
Solanum quitoense Lam. (FC905)	Chila	Cultivated	Skin and subcutaneous tissue	Acne	Fr	Fs	Bt	1
			Blood and cardio-vascular system	High pressure	Fr	If, Jc	Or	2
			Cultural diseases and disorders	Susto, espanto	Lf	If	Bt	1
Solanum tuberosum L. (FC910)	Papa	Cultivated	General ailments with unspecific symptoms	Fever	Ro	Fs	Kn, Pl	4
				Headache	Ro	Fs	Kn, Pl	2
			Digestive system	Stomach cramps	Ro	If	Or	2
				Diarrhoea	Ro	If, Jc, Ml	Or	20
				Gastric ulcers	Ro	If, Ml	Or	6
			Other uses	Diabetes	Ro	Dc, Rt	Or	2
			Infections and infestations	Chickenpox	Ro	Rt	Bt	1
			Reproductive system and reproductive health	Menopause	Fr	Dc	Or	1
			Sensory system	Hearing disorders	Ro	Fs	Pl	1
			Skin and subcutaneous tissue	Burns	Ro	If	Or, Pl	2
			Urinary system	Kidney disorders, emollient, diuretic	Ec, Ro	Dc, If	Or	6
				Prostate disorders	Ro	Dc	Or	1
			**Theaceae**					
Camellia sinensis (L.) Kuntze	Té verde	Cultivated	Metabolic system and nutrition	Weight loss	Lf	If	Or	4
			**Tropaeolaceae**					
Tropaeolum majus L. (FC914)	Capuchina	Cultivated	Cultural diseases and disorders	Tacsho	Lf	Wwm	Or	1
				Aire, malaire	Lf	Wm	Bt	1
Tropaeolum tuberosum Ruiz & Pav. (FC915)	Mashua	Cultivated	Skin and subcutaneous tissue	Feet fungus	Lf	Ml	Pl	1
			Urinary system	Prostate disorders	Fr, Lf	If, Ml	Or	2
			**Typhaceae**					
Typha angustifolia L. ()	Chante	Wild	Digestive system	Intestinal parasites	Lf	Ml	Or	1
			**Urticaceae**					
Pilea microphylla (L.) Liebm. (FC916)	Contrayerba, rumusol, quishquirrinrri	Wild	Muscular-skeletal system	Hernia	Lf	Ml	Pl	1
			Nervous system and mental health	Sadness	Lf	Ml	Or	1
			Urinary system	Hearing disorders	Lf	Wm	Pl	1
Urtica urens L. (FC917)	Ortiga, ortiga negra, ishanga negra	Wild	Reproductive system and reproductive health	Menopause	Lf	If	Or	1
			Other uses	Hair loss	Lf	Dc, Ml	Bt, Pl	3
			Blood and cardio-vascular system	High pressure	Lf	Ml	Or	1
				Hemorrhoids	Lf	Ml	Or	2
			Digestive system	Stomach cramps	Lf	Ml	Or	1
			Muscular-skeletal system	Rheumatism	Lf	Fs, If	Bt, Or, Pl, Wh	4
			Urinary system	Kidney disorders, emollient, diuretic	Lf	Dc	Or	1

			Reproductive system and reproductive health	Menstruation disorders	Ep, Lf, Ro	Dc, If, Mml	Or	23
				Menopause	Ep	If	Or	1
**Verbenaceae**								
Aloysia citriodora Palau (FC918)	Cedrón	Cultivated	Other uses	Hair loss	Lf	If	Or	1
			Digestive system	Stomach infection	Lf	If	Or	1
				Stomach cramps	Lf	If	Or	1
				Diarrhoea	Lf	If	Or	2
			Pregnancy, birth and puerperium	Birth	Lf	If	Or	1
			Reproductive system and reproductive health	Menopause	Lf	If	Or	1
				Fertility	Sd	If	Or	1
			Nervous system and mental health	Insomnia	Lf	If	Or	2
			Blood and cardio-vascular system	High pressure	Lf	If	Or	3
Lantana haughtii Moldenke (FC919)	Sachaorégano	Wild	Digestive system	Stomach cramps	Lf	Fm	Or	1
Verbena litoralis Kunth (FC920)	Verbena	Wild	General ailments with unspecific symptoms	Fever	Ep, Lf	Dc, If, Ml	Bt, Or, Pl	25
				Headache	Lf	Dc	Bt	1
			Pregnancy, birth and puerperium	Abortive	Lf	Dc	Or	1
			Digestive system	Intestinal parasites	Ep	Ml	Or	1
				Stomach cramps	Lf	If	Or	2
				Hepatitis	Lf	Fs, If	Or	4
				Gastric ulcers	Lf	If	Or	1
			Respiratory system	Flu	Lf	If	Or	2
			Infections and infestations	Fleas	Lf	Dc	Bt	1
				Malaria	Lf	Ml	Or	1
				Insect bite	Lf	Ml	Kn	1
				Chickenpox	Lf	Ml	Or	1
			Other uses	Hair loss	Lf	Dc	Bt	1
			Endocrine system	Diabetes	Lf	If	Or	1
**Violaceae**								
Viola odorata L. (FC921)	Violeta	Cultivated	Infections and infestations	UTA, leishmaniasis	Fl	Fs	Pl	1
**Vitaceae**								
Vitis vinifera L. (FC922)	Uva	Cultivated	Digestive system	Indigestive	Fr	Fs	Or	2
				Laxative	Fr	Ddd	Or	1
				Intestinal infection	Fr	Fs	Or	2
			Metabolic system and nutrition	Weight loss	Fr	Fs	Or	1
			Nervous system and mental health	Mental stimulant	Fr	Fs	Or	2
			Reproductive system and reproductive health	Menstruation disorders	Fr	If	Or	1
**Xanthorrhoeaceae**								
Aloe vera (L.) Burm. f. (FC923)	Pencasábila, sábila, aloe	Cultivated	Blood and cardio-vascular system	Hemorrhoids	Lp	Fs, Ml	Kn, Or, Pl	4
			Digestive system	Diarrhoea	Lp	Fs, If, Jc, Ml	Or	28
			General ailments with unspecific symptoms	Fever	Lp	Ml	Kn	1
			Infections and infestations	Insect bite	Lp	Ml	Kn	1
			Other uses	Hair loss	Lp	Fs, If, Ml	Bt, Kn, Or, Pl	20
				Cancer	Lp	If, Ml	Or	4
			Endocrine system	Diabetes	Lp	Ml	Or	1
			Reproductive system and reproductive health	Menstruation disorders	Lp	Ml	Or	1
			Ritual and magic uses	Negative vibes	Lp	Fs	Nn	2
				Bring good luck	Lp	Fs	Nn	2

				Wounds, healing	Lp	Fs	Pl	1
			Skin and subcutaneous tissue	Acne	Lp	Fs, Ml	Bt, Kn, Pl	23
				Feet fungus	Lp	Fs	Pl	5
				Burns	Lp	Fs, Ml	Kn, Pl	7
			Urinary system	Prostate disorders	Lp	Fs	Or	1
			**Zingiberaceae**					
Curcuma longa L.	Cúrcuma	Cultivated	Nervous system and mental health	Insomnia	Fr	Dc	Or	1
Hedychium coronarium J. Koenig (FC924)	Azafrán	Wild	Muscular-skeletal system	Rheumatism	Ep	If	Or	1
			Skin and subcutaneous tissue	Burns	Fr, Lf, Ro	Dc, If, Mml	Bt, Kn, Pl	8
			Other uses	Cancer	Lf	Dc	Or	1
			Urinary system	Prostate disorders	Fr	Dc	Or	1
Renealmia sp. (FC925)	Caña agria	Wild	Urinary system	Prostate disorders	Lf	Ml	Or	1
Zingiber officinale Roscoe (FC926)	Kión	Cultivated	Other uses	Cancer	Ro	Dc	Or	1
			Skin and subcutaneous tissue	Feet fungus	Ro	Dc, Fs	Bt	2
			Blood and cardio-vascular system	Hemorrhoids	Ro	Dc	Or	1
			Digestive system	Diarrhoea	Ro	Ml	Or	3
				Stomach cramps	Ro	If	Or	1
			Metabolic system and nutrition	Weight loss	Ro	Dc, If, Ml	Or	6
			Nervous system and mental health	Insomnia	Ro	If	Or	1
			Respiratory system	Flu	Ro	Dc, Fs, If	Or	8
				Cough	Ro	Fm, If	Or	2
			Sensory system	Visual disorders	Ro	If	Bt	1
			Urinary system	Prostate disorders	Ro	If	Or	1
				Kidney disorders, emollient, diuretic	Ro	Fs	Or	1

Plant parts

Ap Aerial part, *Bd* Bud, *Bk* Bark, *Ec* Exocarp, *Ep* Entire plant, *Fl* Flower, *Fr* Fruit, *Lf* Leaf, *Lp* Leaf pulp, *Lx* Látex, *Ro* Root, *Sd* Seed, *Sp* Spine, *Ss* Stigmas and styles, *St* Stem

Preparation form

Dc Decoction, *Dd* Dried, *Fm* Fermented, *Fs* Fresh, *If* Infusion, *Jc* Juice, *Ml* Molten, *Rt* Rotten, *Wm* Warmed

Mode of administration

Bt Baths, *En* Enema, *Ew* Eyewash, *Gg* Gargle, *In* Inhaled, *Kn* Kneaded, *Nn* None, *Oi* Oil, *Or* Oral, *Pl* Plaster, *Vp* Vapours, *Wh* Whip

All voucher specimens were collected by Corroto, Mostacero and Mejía

Table S2. Medicinal plants used in the city of Chachapoyas, in the tropical montane forests of northern (Peru).

**Cultural Diseases and Disorders**	**Explanation**
Aire/Malaire	It is a strong headache, together with intense muscle sprains. “Malaire” (which can be literally traduced by “malevolent wind”), is said to be caused by weird air streams, warm or cold, coming down from the mountains, and affecting people by direct contact (De la Cruz *et al*., 2007).
Susto/Espanto	It is an intense fright, affecting children and adults, caused by spirits from the wild (mountains and rivers), affecting and taking control over people (De la Cruz *et al*., 2007).
Antimonia/Gentil/Viejo/Antiguo	Bone or skin infection due to contact with bones from pre-Inca tombs called "morada de gentiles"
Tacsho	

	It is a gaze or evil eye of a dead person, generally in children, in such a way that the belief is that, in a family, a person when dying, out of affection, can leave a glance at the boy or girl, and in that case they have to be healed through the liberation of the spirit of the dead.
Tijte	Skin irritation caused by coming into contact with certain species of plants with cross-leaf growth.
